# Absence of Batf3 reveals a new dimension of cell state heterogeneity within conventional dendritic cells

**DOI:** 10.1016/j.isci.2021.102402

**Published:** 2021-04-15

**Authors:** Samuel W. Lukowski, Inga Rødahl, Samuel Kelly, Meihua Yu, James Gotley, Chenhao Zhou, Susan Millard, Stacey B. Andersen, Angelika N. Christ, Gabrielle Belz, Ian H. Frazer, Janin Chandra

**Affiliations:** 1 The Institute for Molecular Bioscience, The University of Queensland, 4067, QLD, Australia; 2 The University of Queensland Diamantina Institute, The University of Queensland, Woolloongabba, QLD 4102, Australia; 3 Mater Research, Translational Research Institute, Woolloongabba, 4102 QLD, Australia; 4 The Walter and Eliza Hall Institute of Medical Research, Parkville, VIC, 3052, Australia; 5 Department of Medical Biology, University of Melbourne, Parkville, VIC, 3010, Australia

**Keywords:** Immunology, Cell Biology, Transcriptomics

## Abstract

Conventional dendritic cells (cDCs) are traditionally subdivided into cDC1 and cDC2 lineages. Batf3 is a cDC1-required transcription factor, and we observed that *Batf3*−/− mice harbor a population of cDC1-like cells co-expressing cDC2-associated surface molecules. Using single-cell RNA sequencing with integrated cell surface protein expression (CITE-seq), we found that *Batf3*−/− mitotic immature cDC1-like cells showed reduced expression of cDC1 features and increased levels of cDC2 features. In wild type, we also observed a proportion of mature cDC1 cells expressing surface features characteristic to cDC2 and found that overall cDC cell state heterogeneity was mainly driven by developmental stage, proliferation, and maturity. We detected population diversity within *Sirpa*+ cDC2 cells, including a *Cd33*+ cell state expressing high levels of *Sox4* and lineage-mixed features characteristic to cDC1, cDC2, pDCs, and monocytes. In conclusion, these data suggest that multiple cDC cell states can co-express lineage-overlapping features, revealing a level of previously unappreciated cDC plasticity.

## Highlights

• Single-cell proteogenomics identifies additional layers of DC heterogeneity
• cDC diversity is driven by proliferation, developmental stage, and maturation
• Lack of *Batf3* increases cDCs with lineage-mixed features
• Sox4+ cDCs represent a cell state of lineage-mixed features

## Graphical abstract

**Figure:**
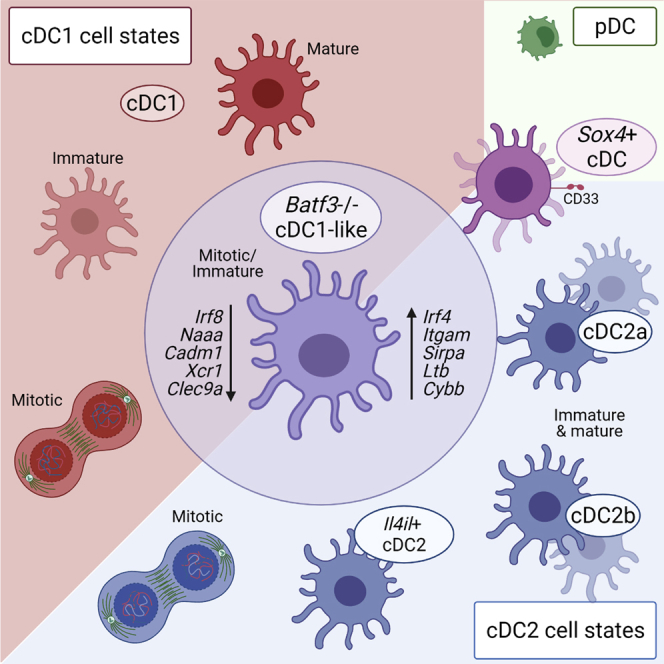

## Introduction

Conventional dendritic cells (cDCs) play a central role in bridging innate and adaptive immune responses due to their specialization in antigen presentation and activation of T cells. The current terminology distinguishes cDC1 and cDC2 lineages based on differential ontogeny, expression of surface features, requirement for transcription factors, and function. cDC1 cells are highly specialized in cross-presentation and activation of cytotoxic T cells, whereas cDC2 cells are primarily driving helper T responses (Wculek et al., 2020).

The development of cDCs from precursor CD34+ cells is driven by specific transcription factors shared between mouse and human (Schultze et al., 2019), and cDC subtype divergence is largely driven by interferon-regulatory factors 4 and 8 (IRF4/IRF8) (Murphy et al., 2016; Seillet and Belz, 2013). cDC1 in mice express CD8a and/or CD103, and CD141 in humans. Across species, cDC1 are classified as IRF8+ or Batf3-dependent cDCs, and cDC1 development and maintenance depends on transcription factors IRF8, ID2, and Batf3. While IRF8 and ID2 are indispensable for lineage commitment, Batf3 is required for sustained IRF8 autoactivation and subsequently for the maintenance of cDC1 (Seillet et al., 2013; Grajales-Reyes et al., 2015). Function-conveying features of cDC1 are XCR1 and CLEC9A. cDC2 in mice are CD11b+ CD172a+, and CD4+ when residing in lymphoid tissues, whereas in humans, cDC2 express CD1c. Across species, cDC2 are classified as IRF4+ or Batf3-independent, and in human and mice, transcription factors driving cDC2 commitment include IRF4, NOTCH2, Rbp-J, and KLF4. In addition, our understanding of cDC2 heterogeneity is steadily increasing.

Recent advances in high-dimensional single-cell technologies have challenged the canonical cDC classification. Several groups utilizing single-cell omics technologies described unprecedented heterogeneity within cDCs in both humans and mice. The current consensus is that cDC1 are relatively homogeneous, but cDC2 are heterogeneous in phenotype and function (Brown et al., 2019; Villani et al., 2017). Recently, cDC2 were shown to comprise functionally distinct subpopulations expressing either TBET or RORGT, transcription factors commonly associated with T cells and innate lymphoid cells (Brown et al., 2019). Additionally, transitional dendritic cells (DCs) and DC3 have been described in both mouse and human, which share monocyte features and are efficient antigen presenters (Leylek et al., 2019). Single-cell RNA sequencing (scRNA-seq) analysis of cDCs has enabled the identification of subpopulation-specific gene signatures and advanced our understanding of cDC heterogeneity. Despite this, accurate cDC2 classification remains unresolved due to the reduced ability to detect expression of cDC2-characteristic transcripts such as *Itgam* (CD11b), *Cd4,* and *Irf4* (Stoeckius et al., 2017).

We previously reported that the acquisition of a cDC1 phenotype and function depends on Batf3 and that in the absence of Batf3, a residual population of CD8+ cDCs co-expresses surface molecules characteristic to both cDC1 and cDC2 lineages (Chandra et al., 2017). Consequently, we sought here to explore cell state plasticity of cDCs in steady state and in the absence of Batf3 using multiple single-cell technologies including conventional and imaging flow cytometry, and scRNA-seq with integrated cell surface protein expression. This enabled us to identify cDC cell states co-expressing mixed cDC phenotypic and transcriptomic signatures. We identified MHCII+ CD11c+ cells expressing both CD11b and CD8a in steady state, which were increased in *Batf3*−/− mice. We demonstrate that mature classical cDC1 cells are nearly absent in *Batf3*−/− mice, and that a population of mitotic cDC1-like cells remained immature and likely non-functional due to diminished expression of the classical cDC1 markers *Tlr3*, *Xcr1,* and *Clec9a*. This population further expressed increasing levels of cDC2 genes, which supports the notion by others of a possible phenotypic plasticity between different DC subtypes (Grajales-Reyes et al., 2015; Lau et al., 2018). Our data further revealed a population of *Sirpa*+ *Sox4*+ *Cd33*+ cDCs expressing lineage-mixed features of cDC1, cDC2, pDCs, pre-DCs, and monocytes in both wild-type (WT) and *Batf3*−/− mice. cDCs with lineage-mixed phenotypes have recently caught attention in multiple other studies in both human and mice (Brown et al., 2019; Leylek et al., 2019; Bosteels et al., 2020; Bourdely et al., 2020; Cytlak et al., 2020). The *Sirpa*+ *Sox4*+ *Cd33*+ cDCs lineage-mixed cell state we describe here lacks expression of toll-like receptor (TLR) genes and genes indicating cDC1 functions, whereas it expresses the highest levels of *Lag3* among cDCs. Our data further demonstrate differential expression of co-inhibitory molecules across different cDC cell states, which may assist cDC cell state-specific target prioritization for checkpoint inhibition.

## Results

### Identification of cDCs that express both cDC1- and cDC2-characteristic surface features

**Figure 1:**
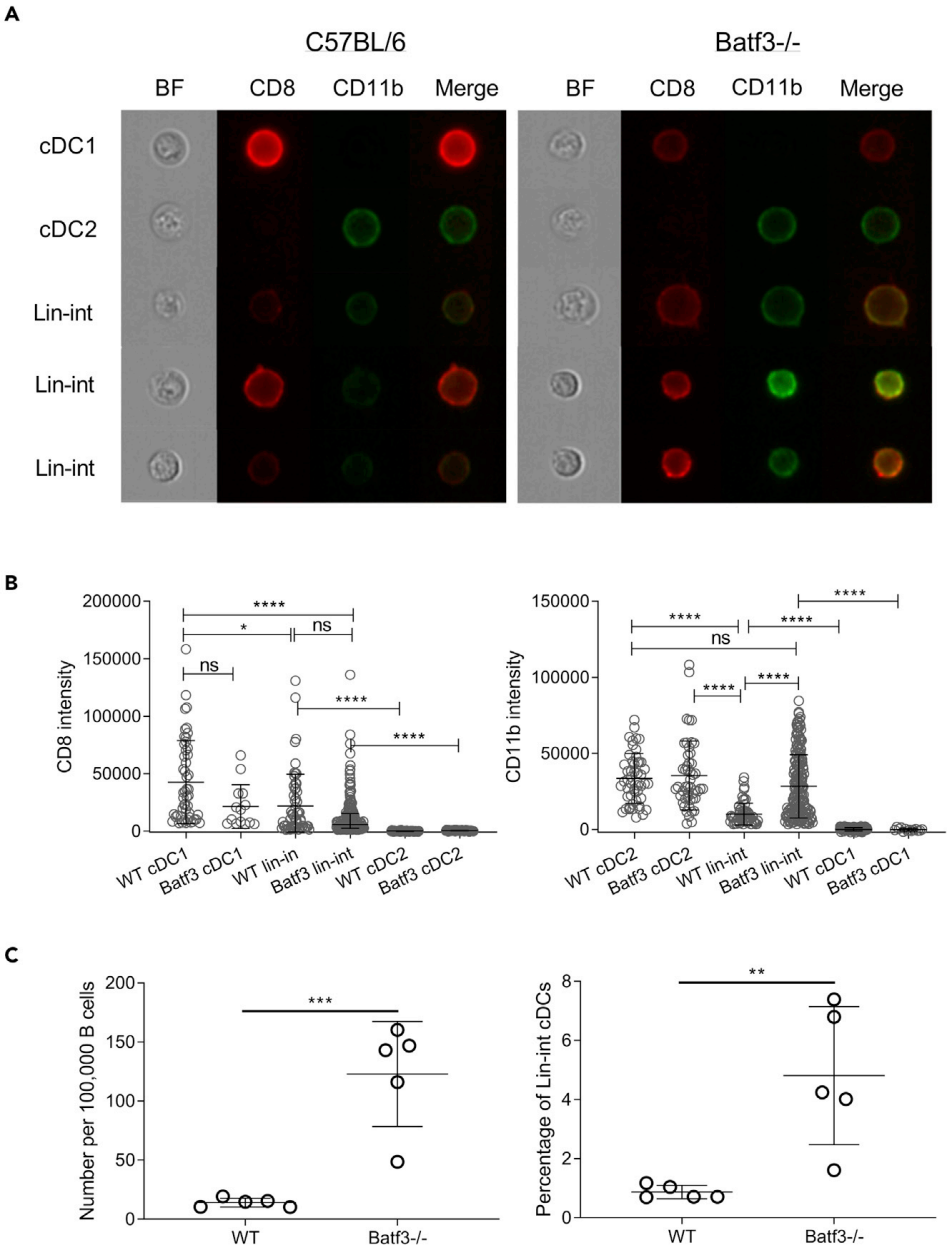
Absence of *Batf3* leads to enrichment of lineage-intermediate cDCs (A and B) MHCII+ CD11c+ splenic cDCs of C57BL/6 WT and *Batf3−/−* mice were assessed for expression of CD8 and CD11b by imaging flow cytometry. (A) Pre-gated to MHCII+ CD11c+ cells, cDC1 cells were identified as CD8+CD11b−, cDC2 cells as CD8−CD11b+, and lineage-intermediate cDCs as CD8+CD11b+ (Figure S1). BF, brightfield; merge, overlay of CD8 and CD11b. (B) Intensities of CD8 and CD11b was compared between C57BL/6 WT and *Batf3−/−* cDC lineages. Each data point represents an individual cell with mean and interquartile range indicated. (C) cDCs in splenocytes of C57BL/6 WT and Batf3-/- mice (n=5) were immunoprofiled using conventional flow cytometry. The number of CD8+CD11b+ cDCs per 100,000 B cells was compared. Statistical significance was determined using one-way ANOVA followed by Tukey's multiple comparison test. ∗p < 0.05, ∗∗p < 0.01, ∗∗∗p < 0.001, ∗∗∗∗p < 0.0001. Shown is one of two independent experiments. See also Figure S1.

Batf3 is required for continued autoactivation of IRF8 in cDC precursors, allowing commitment of these cells to the cDC1 lineage (Grajales-Reyes et al., 2015). However, this commitment can also be achieved in the absence of Batf3 by infection-associated induction of IL-12, which promotes expression of Batf, enabling cDC1 development in *Batf3*−/− mice (Tussiwand et al., 2012). It is therefore not surprising that cDC1 development in *Batf3*−/− animals can vary with the health of the studied animals (Mott et al., 2015). Using a multiparameter flow cytometry approach, we previously established that *Batf3*−/− mice harbor a residual population of CD8+ cDC cells which co-express increasing levels of cDC2-characteristic surface molecules CD11b, CD172a, and CD4, but lack IRF8 (Chandra et al., 2017). However, technical artifacts in conventional flow cytometry might explain this finding. Therefore we used imaging flow cytometry to confirm the existence of cDCs that express both cDC1- and cDC2-characteristic surface features. T- and B cell-depleted splenocytes were used to initially identify CD11c+ MHCII+ cDCs (Figure S1), which were subsequently analyzed for surface expression of CD11b and CD8. Classical cDC1 and cDC2 were identified as single positive for CD8 or CD11b, respectively (Figures 1A and S1). We further identified lineage-intermediate (lin-int) cDCs co-expressing CD8 and CD11b on the cell surface in both WT and *Batf3*−/− mice. We compared the expression level of CD8 and CD11b between lineage-intermediate DCs, cDC1 and cDC2 and found that WT and *Batf3*−/− lineage-intermediate cDCs expressed significantly more CD8 when compared with CD8− cDC2, but less than CD8+ cDC1 (Figure 1B). Similarly, lineage-intermediate DCs expressed significantly more CD11b when compared with CD11b− cDC1, and less when compared with CD11b+ cDC2 (Figure 1B). These CD8+ CD11b+ cDCs comprised a higher abundance in *Batf3−/−* animals than in wild-type animals (Figure 1C).

### Cell state plasticity of splenic cDCs is driven by ontogeny, cell cycle, and maturation

Of note, our previous data indicated that CD8+ CD11b+ cDCs demonstrated a continuum of CD8 and CD11b expression (Chandra et al., 2017) suggesting that these cells do not represent a committed cell lineage, but more likely demonstrate cell state plasticity. To uncover the transcriptional basis of this phenomenon, we carried out microfluidic droplet-based scRNA-seq of purified splenic cDCs in combination with barcoded cDC-specific antibodies, referred to as cellular indexing of transcripts and epitopes by sequencing (CITE-seq) (Stoeckius et al., 2017). After T and B cell depletion, cDCs were sorted to purity as live singlet CD11c+ MHCII+ cells (Figure S2). We sequenced RNA from 13,943 WT and 13,750 *Batf3*−/− cDCs and obtained ∼30,000 reads per cell, from a median number of 2,352 genes per cell. After pre-processing and quality control, the data from 12,224 WT and 12,119 *Batf3*−/− cells underwent log normalization and uniform manifold approximation and projection (UMAP) dimensionality reduction.

**Figure 2:**
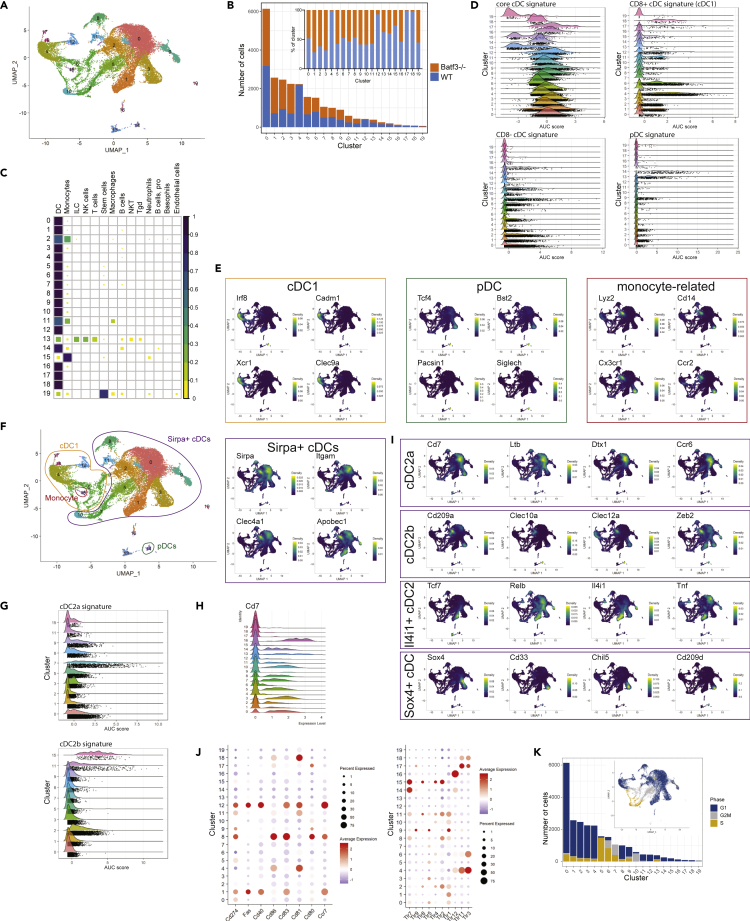
Splenic cDC compartment consists of heterogeneous cell states of cDC1 and cDC2 Sorted MHCII+ CD11c+ splenic DCs of C57BL/6 and *Batf3*−/− mice (see Figure S2) were incubated with Total-Seq antibodies and sequenced using the 10X Genomics droplet-based sequencing platform. Application of the Seurat pipeline resulted in 20 clusters at resolution 0.5. (A) UMAP depicting clusters based on Louvain algorithm using the FindNeighbors function in Seurat. (B) Proportion and number of cells assigned to WT and *Batf3−/−* genotypes. (C) SingleR prediction of clusters using the ImmGen database-identified DC clusters and non-DC contaminations. (D) Gene set enrichment analysis (GSEA) using DC gene signatures from (Miller et al., 2012) and the AUCell package (see Table S2). (E) Density plots of key canonical DC and monocyte-delineating features using the Nebulosa package (Jose Alquicira-Hernandez, 2020). (F) Definition of cluster groups for cDC1, *Sirpa*+ DCs, pDCs, and monocytes. (G) GSEA of *Sirpa*+ DC clusters and monocytes (C15) using cDC2a and cDC2b gene signatures from Brown et al. (2019) and the AUCell package (see Table S2). (H) Ridgeplot of *Cd7* expression. (I) Density plots of key genes delineating *Sirpa*+ cDC clusters using the Nebulosa package. (J) Expression of maturation-associated and TLR genes. (K) Distribution and proportion of cells assigned to cell cycle phases based on the expression of gene signatures of the G1, S, and G2/M phases (see Table S5). See also Table 1, Figures S3 and S4, Tables S1, S3, and S4.

Unbiased graph clustering using the Seurat pipeline (Butler et al., 2018) at a default resolution of 0.5 identified 20 clusters (C) of the sorted CD11c+ MHCII+ cells (Figure 2A). To determine the differences between WT and *Batf3−/−* cells, composition analysis by genotype was undertaken, and showed that C4, C12, C17 and C18 were almost exclusively represented by WT, whereas no *Batf3−/−*-specific cluster was found (Figure 2B). We analyzed differentially expressed genes (DEGs) between the WT and *Batf3−/−* genotypes for each cluster. The majority of clusters comprised similar numbers of cells from both genotypes and, with the exception of C4, C6, and C12, revealed only *Batf3* as a DEG (Table S1). Given the overall similarity of the WT and the *Batf3*−/− clusters, we proceeded to annotate cluster identities from the combined dataset.

Using SingleR (Aran et al., 2019) and the integrated Immunological Genome Project (ImmGen) data (Heng et al., 2008; Miller et al., 2012) to compare the single-cell transcriptional features with immune cell expression profiles, C13 were predicted to be NK/NKT/ILC/T cells, C15 to be monocytes, C19 to be stem cells, and the remaining 17 clusters to be DCs (Figure 2C). Of note, C2 and C11 were predicted to be DCs with high confidence, but were also moderately enriched with a monocyte signature. With the exception of the C13 cluster, no gene expression of *Klrb1a*, *Cd19,* or *Cd3e* was detected, demonstrating the absence of NK, B and T cells within the DC clusters (Figure S3A). All DC clusters expressed MHC class II genes and *Itgax* (CD11c) (Figure S3B). Using the DC lineage-specific cell signatures published by the Immgen group (Miller et al., 2012) (Table S2), we tested each cluster for enrichment of signatures of core cDCs, CD8+ cDCs (cDC1), CD8− cDCs, and pDCs using the *AUCell* package (Aibar et al., 2017). Consistent with the SingleR analysis, C13, C15, and C19 were not enriched for a core cDC signature, whereas all other clusters aligned (Figure 2D). Cells of C14 displayed the highest pDC area under curve (AUC) score. C4, C6, C12, C17, and C18 had the highest cDC1 AUC scores. Of note, the Immgen CD8− cDC signature only partially aligned with the remaining DC clusters and was enriched in the monocyte cluster C15, indicating that this signature is not cDC2 specific. However, this was expected with respect to our current knowledge of heterogeneity within CD8− cDCs.

Expression analysis of key canonical DC and monocyte genes further supported the identity of pDCs, cDC1, cDC2, and monocytes (Figure 2E). Expression of *Sirpa*, traditionally associated with a cDC2 phenotype, was the most reliable identifier of a non-cDC1 cDC2 phenotype (Figure 2E). Based on these analyses, we broadly subgrouped DC clusters into cDC1, *Sirpa*+ DCs and pDCs (Figure 2F).

The *Sirpa*+ DC clusters C2, C3, and C11 additionally displayed features characteristic of monocytes including *Cd14* and *Ccr2* (Figure 2E). Recent studies have identified at least two subtypes of *Sirpa*+ DCs. Brown et al. (2019) recently characterized cDC2a and cDC2b populations by the differential expression of T-bet and RORγt, respectively, with cDC2b displaying monocyte features. We used the previously published cDC2a- and cDC2b-specific gene signatures (Brown et al., 2019) (Table S2) to identify the presence of these cells within *Sirpa*-expressing clusters and compare these signatures to monocytes (C15). Among *Sirpa*+ cDCs, C7 displayed the highest cDC2a AUC score, whereas C2 and C11 displayed the highest cDC2b AUC scores (Figure 2G). Indeed, the monocyte cluster C15 expressed the highest cDC2b AUC score overall (Figure 2G), but previous studies have shown that despite expression of monocyte features, cDC2b cells are not monocyte-related (Brown et al., 2019). The remaining *Sirpa*-expressing clusters displayed moderate cDC2a AUC scores.

Furthermore, differential gene expression (DGE) analysis (Table S3) between clusters (one versus remaining) revealed *Cd7* as a transcript feature dominantly expressed by cDC2a clusters (Figure 2H). We propose that *Cd7* in combination with *Sirpa*, MHCII, and *Itgax* transcripts could therefore identify cDC2a. *Cd7* expression by cDC2a cells has been observed by others in DC single-cell sequencing experiments (Brown et al., 2019; Bosteels et al., 2020). We further note that whereas no *Cd7* expression was observed in cDC1 clusters, the level of expression varied within cDC2a clusters, and was highest expressed in C0, suggesting that *Cd7* expression is regulated.

In addition to the cDC2a clusters (C0, C5, C7, C9, C8, and C10) and cDC2b clusters (C2 and C11), we identified two main *Sirpa*+ cDC clusters (C1 and C3) that expressed unique DEGs (Table S3). C1 was characterized by expression of genes associated with TNF signaling as well as the highest expression of *Relb* and *Tcf7,* whereas lacking expression of *Itgam, Cd7,* or any cDC2b features such as *Cd14* and *Ccr2* (Figure 2I). C1 also expressed *Ccr7*, *Cd40*, *Fas,* and the immune-suppressive genes *Cd274* (PD-L1) (Figure 2J) and *Il4i1.* Based on expression of *Fabp5*, *Spint2,* and *Il4i1*, this cluster aligned with a migratory cDC2 phenotype described by others (Bosteels et al., 2020). However, we note that C1 cells were distinct from other *Ccr7*-expressing cDC2a (C8) and cDC2b (C11) cells in our dataset (see DEGs between C1, C8, and C11 in Table S4). We annotated C1 as *Il4i1*+ cDC2 as *Il4i1* was a highly significant DEG (Figure 2I). The other distinct cDC cluster C3 highly expressed *Sox4*, *Cd33,* and *Tcf4* (Figures 2I, Table S3). Together with expression of *Sirpa, Cd8a, Cd14*, *Ccr2,* and low levels of *Siglech,* this cluster displayed similarities to a murine *Siglech*+ DC phenotype described by others, sharing pDC and cDC2 characteristics, which were termed transitional DCs (Leylek et al., 2019; Brown et al., 2019), and similar populations have also been described in different contexts (Bar-On et al., 2010; Schlitzer et al., 2011; Rodrigues et al., 2018). Based on the most significant DEG, we annotated this cluster as *Sox4*+ cDC (Figure 2I).

To test whether proliferation contributed to cluster heterogeneity, we predicted the cell cycle phase of each individual cell using S and G2M signature genes defined by Tirosh and colleagues (Tirosh et al., 2016) (Table S5) and identified C5, C6, C7, C10, and C19 as mitotic (Figure 2K).

DGE analysis between clusters revealed a prominence of mitochondrial gene expression in C9 and C17 (Suppl. File 1, Data S3, Figure S3C), and these clusters simultaneously expressed lower levels of MHCII genes and *Itgax* (Figure S3B). Cluster C18 was enriched in ribosomal subunit genes (Table S3, Figure S2C). After removing low-quality cells in the initial quality control (see transparent methods) and before PCA and clustering, we further analyzed the data for expression of genes associated with apoptosis, and no cluster expressed particularly increased levels of apoptosis genes (Figure S3D), however, C9 and C17 had markedly lower unique molecular identifier (UMI) counts (Figure S3E).

We constructed a knowledge-informed list of canonical genes characteristic to cDC1, cDC2, and pDCs (Tomasello et al., 2018). This list, consistent with the cDC1 AUC score, revealed high expression of cDC1-associated genes in C4, C6, C12, C17, and C18 (Figure S4). Analyzing DEG-enriched pathways (Table 1) and maturation features (Figure 2J), we identified C1, C8, C11, and C12 as clusters with increased maturity based on expression of *Ccr7*, co-stimulatory molecules (Figure 2J), or genes associated with TNF signaling. Analysis of TLR genes further enabled cDC lineage annotation (Figure 2J). Using the information from each of these analyses, we annotated clusters according to cell lineage and cell state (Table 1).

**Table 1:** Cluster annotation (related to Table S3)

Cluster	Cell lineage	Cell state	GO Cellular Component enrichment
0	*Sirpa*+ cDC2a	Immature, phagocytic	Secretory granule lumen (GO:0034774), cytoplasmic vesicle lumen (GO:0060205), tertiary granule (GO:0070820)
1	*Sirpa*+ *Il4i1*+ cDC2	Mature	CD40 receptor complex (GO:0035631), tertiary granule (GO:0070820), specific granule (GO:0042581),
2	*Sirpa*+ cDC2b	Immature, phagocytic	Tertiary granule (GO:0070820), tertiary granule membrane (GO:0070821), azurophil granule (GO:0042582)
3	*Sirpa*+ *Sox4*+ cDC	Immature, phagocytic	Tertiary granule (GO:0070820), tertiary granule membrane (GO:0070821), specific granule (GO:0042581)
4	cDC1	Immature, phagocytic	Lysosomal lumen (GO:0043202), lysosome (GO:0005764), phagocytic vesicle (GO:0045335)
5	*Sirpa*+ cDC2a	Mitotic	Nuclear chromosome part (GO:0044454), chromosome, telomeric region (GO:0000781), nuclear chromosome, telomeric region (GO:0000784)
6	cDC1	Mitotic, immature	Mitochondrial inner membrane (GO:0005743), nuclear chromosome part (GO:0044454), mitochondrial proton-transporting ATP synthase complex (GO:0005753)
7	*Sirpa*+ cDC2a	Mitotic	Nuclear chromosome part (GO:0044454), spindle (GO:0005819), chromosome, centromeric region (GO:0000775)
8	*Sirpa*+ cDC2a	Mature	P-body (GO:0000932), fibrillar center (GO:0001650), nucleolar part (GO:0044452)
9	*Sirpa*+ cDC2 (mixed)	Mitochondrial	Nuclear body (GO:0016604), nuclear speck (GO:0016607), chromatin (GO:0000785),
10	*Sirpa*+ cDC2a	Mitotic	Spindle (GO:0005819), chromosome, centromeric region (GO:0000775), condensed chromosome, centromeric region (GO:0000779)
11	*Sirpa*+ cDC2b	Mature	Tertiary granule (GO:0070820), ficolin-1-rich granule (GO:0101002), lysosomal lumen (GO:0043202)
12	cDC1	Mature	CD40 receptor complex (GO:0035631), actin cytoskeleton (GO:0015629), cytoskeleton (GO:0005856)
13	NK/ILC/T		Cytosolic ribosome (GO:0022626), T cell receptor complex (GO:0042101), cytosolic part (GO:0044445)
14	PDCs		Lysosomal lumen (GO:0043202), lysosome (GO:0005764), specific granule membrane (GO:0035579)
15	Monocytes		Tertiary granule (GO:0070820), focal adhesion (GO:0005925), specific granule (GO:0042581)
16	Mixed identity		Secretory granule lumen (GO:0034774), ficolin-1-rich granule lumen (GO:1904813), cytoplasmic vesicle lumen (GO:0060205)
17	cDC1	Mitochondrial	Mitochondrial inner membrane (GO:0005743), mitochondrial respiratory chain complex I (GO:0005747), nuclear speck (GO:0016607)
18	cDC1	Ribosomal	Cytosolic ribosome (GO:0022626), cytosolic part (GO:0044445), cytosolic large ribosomal subunit (GO:0022625)
19	Stem cells		Nuclear chromosome part (GO:0044454), spindle (GO:0005819), mitochondrion (GO:0005739)

**Figure 3:**
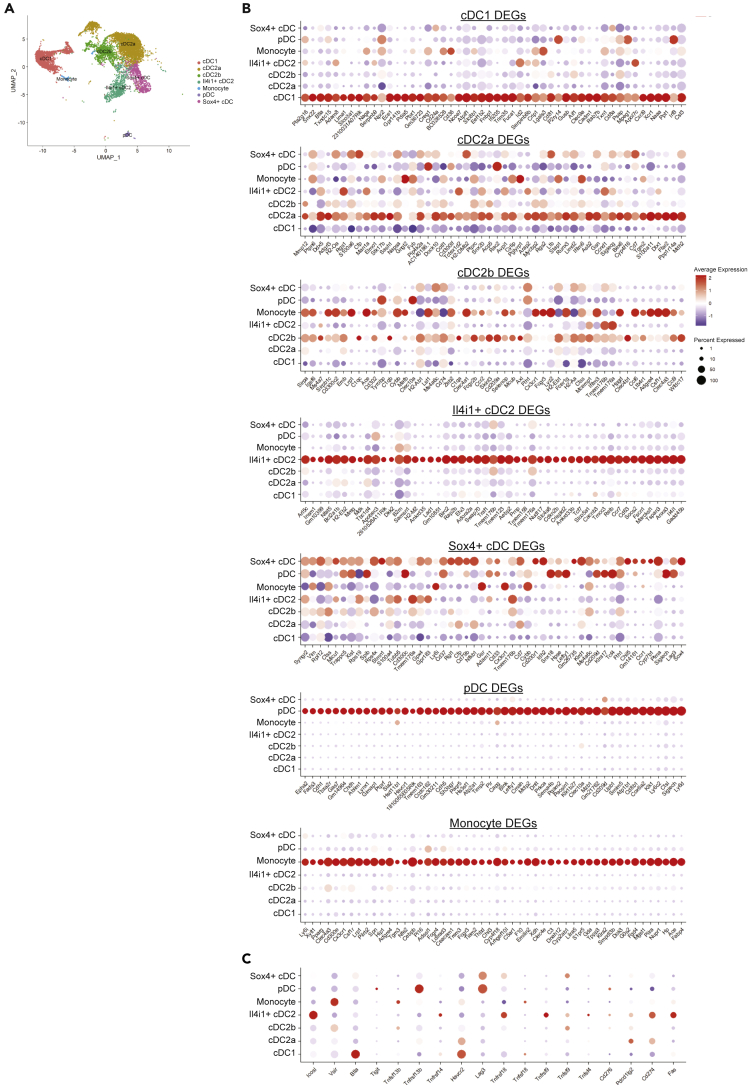
Diversity in *Sirpa*+ DCs is less distinct than diversity between other DC lineages (A) Clusters defined by active cell cycle or metabolic activity were excluded, and metaclusters corresponding to cDC1, cDC2a, cDC2b, *Il4i1*+ cDC2m *Sox4*+ cDC, pDC, and monocytes were established. (B) Dotplots showing the top 50 DEGs of each metacluster (see Table S6). (C) Dotplots showing expression of immune checkpoint genes in each metacluster.

To enable identification of key genes differing between core WT DC clusters, we isolated WT cells and removed clusters characterized by confounding cellular processes such as proliferation (C5, C6, C7, C10) and high expression of mitochondrial (C9, C17) and ribosomal genes (C18), which would hinder identification of key steady state genes between DC clusters. We then built metaclusters for cDC1 (immature C4 + mature C12), cDC2a (immature C0 + mature C8), and cDC2b (immature C2 + mature C11) (Figure 3A). We performed DGE analysis between these core cDC1 and cDC2 clusters as well as *Il4i1*+ cDC2 (C1), *Sox4*+ cDCs (C3), pDCs (C14), and monocytes (C15) (Table S6). cDC1, *Il4i1*+ cDC2, pDC, and monocytes displayed unique gene signatures, whereas DEGs of cDC2a, cDC2b, and *Sox4+* cDC were less clearly defined (Figure 3B). Many cDC2a DEGs were also expressed in *Sox4+* DC, including *Mdh2*, *Cd7*, *Siglecg,* and *Runx3*, suggesting a partial overlap. cDC2b DEGs were largely expressed by monocytes. A significant number of *Sox4+* cDC DEGs were also highly expressed in pDCs, such as *Lag3*, *Siglech*, *Tcf4*, *Spib,* and *Cd37*.

As myeloid cells have become an attractive target for immune checkpoint inhibition therapy to increase their priming capacity, we analyzed the expression of immune checkpoint genes across metaclusters (Figure 3C). This analysis identified that cDC1 cells are the main DCs expressing *Btla* and *Havcr2* (encoding TIM-3), *Il4i1*+ cDC2 express high levels of *Icosl*, whereas monocytes express *Vsir* (encoding VISTA). Interestingly, both pDCs and *Sox4*+ cDCs expressed the highest levels of *Lag3*.

### Simultaneous detection of transcript and epitope enables confident cDC lineage identification

**Figure 4:**
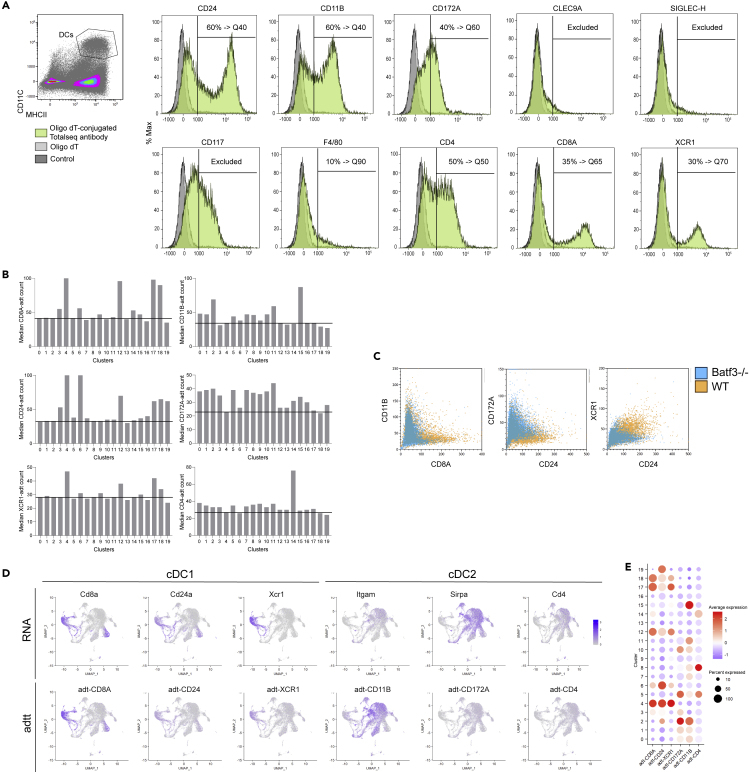
Total-Seq antibodies verify cDC1 and cDC2 identity (A) Verification of Total-Seq antibody binding. Total-Seq antibodies were hybridized with a fluorochrome-conjugated dT probe and subsequently incubated with C57BL/6 splenocytes together with fluorochrome-conjugated antibodies against CD11c and MHCII. Cells were pre-gated on CD11c+ MHCII+ DCs and analyzed for binding of Total-Seq antibodies. Positive signal for Total-Seq antibodies were used to determine thresholds for bioinformatics analysis of adt signals. Total-Seq antibodies where specific staining could not be confirmed were excluded from further analysis of CITE-seq data. (B) Adt sequencing data of each cluster was converted to FSC files using the flowCore package and analyzed using flow cytrometric BD Kaluza analysis software. Shown are median adt tag counts for each cluster. Horizontal line represents background, based on cDC2 cluster C0 for cDC1 features CD8A, CD24, and XCR1, and based on cDC1 cluster C4 for cDC2 features CD11b, CD172A, and CD4. (C) Adt data of cDC1 features CD8A, CD24, and XCR1 or cDC2 features CD11B and CD172A were compared between WT and *Batf3−/−* genotypes. (D) Featureplots of RNA and corresponding thresholded adt counts. (E) Dotplot of thresholded adt counts for each cluster.

Although a cDC1-characteristic gene transcript signature is clearly identified, confident classification of cDC2 is more challenging, as transcription of key cDC2 surface molecules, such as *Itgam* (CD11b) and *Cd4,* is limited (Stoeckius et al., 2017; Peterson et al., 2017). Although it is possible to partly overcome this challenge by recovering single-cell gene expression signals, for example, by using kernel density estimation algorithms implemented in *Nebulosa* (Alquicira-Hernandez and Powell, 2021), parallel detection of protein expression is required to compare transcriptomic and proteomic data. We integrated transcript and epitope multimodel analysis by adding barcoded antibodies specific to DC-characteristic surface molecules. Binding of the barcoded antibodies was confirmed using an oligo-dT probe conjugated to a fluorochrome dye and flow cytometry analysis (Figure 4A). Antibody-derived tag (adt) counts of the complete dataset, each genotype, and each cluster were converted to flow cytometry standard (FCS) files. Analysis of the median adt value for each cluster revealed that each Total-Seq antibody produced a substantial background read count (Figure 4B). This is in line with observations by others when using high antibody concentrations as recommended in suppliers' protocols (Buus et al., 2021). Comparing the characteristic features of cDC1 (CD8A, CD24, XCR1) against those of cDC2 (CD11B, CD172A, CD4) in this way revealed that the expression of these markers was largely restricted to a single-cell type (Figure 4B). In line with previous analysis, the cDC1 clusters C4, C6, C12, C17, and C18 expressed increased levels of CD8A and CD24, whereas the cDC2 clusters C0, C1, C2, C5, and C7–C11 expressed increased levels of CD11B and CD172A (Figure 4B).

Visualization of the adt expression using a conventional FCS dotplot of CD8A versus CD11B or CD24 versus CD172A did not allow confident delineation of cDC1 and cDC2 clusters (Figure 4C); however, comparison between WT and *Batf3−/−* genotypes revealed that a population of CD24+ XCR1+ cells indicative of cDC1 cells was missing in *Batf3*−/− mice (Figure 4C).

A positive adt count for each antibody in each cluster suggested a level of non-specific binding. Informed by the percentage of positive staining in the flow cytometry validation data using the oligo-dT probe described in Figure 4A, we determined adt count thresholds above which we considered the adt signal specific, and all values below this threshold were set to 0. After thresholds were applied, the expression of *Cd8a* transcript and CD8A epitope correlated strongly (Pearson's correlation, r = 0.75; Figure S3F). We detected higher levels of adt-CD11B protein expression compared with the corresponding gene *Itgam*, indicating a substantial benefit to the simultaneous detection of CD11B as an important cDC2 feature (Figure 4D). In contrast, adt detection of CD24, XCR1, CD172A, and CD4 was weaker, when compared with the corresponding *Cd24a*, *Xcr1*, *Sirpa,* and *Cd4* RNA detection. The combination of both RNA and adt protein data verified the identity of cDC1 and cDC2 clusters (Figures 4D and 4E).

### Residual cDC1-like cells in *Batf3−/−* mice

**Figure 5:**
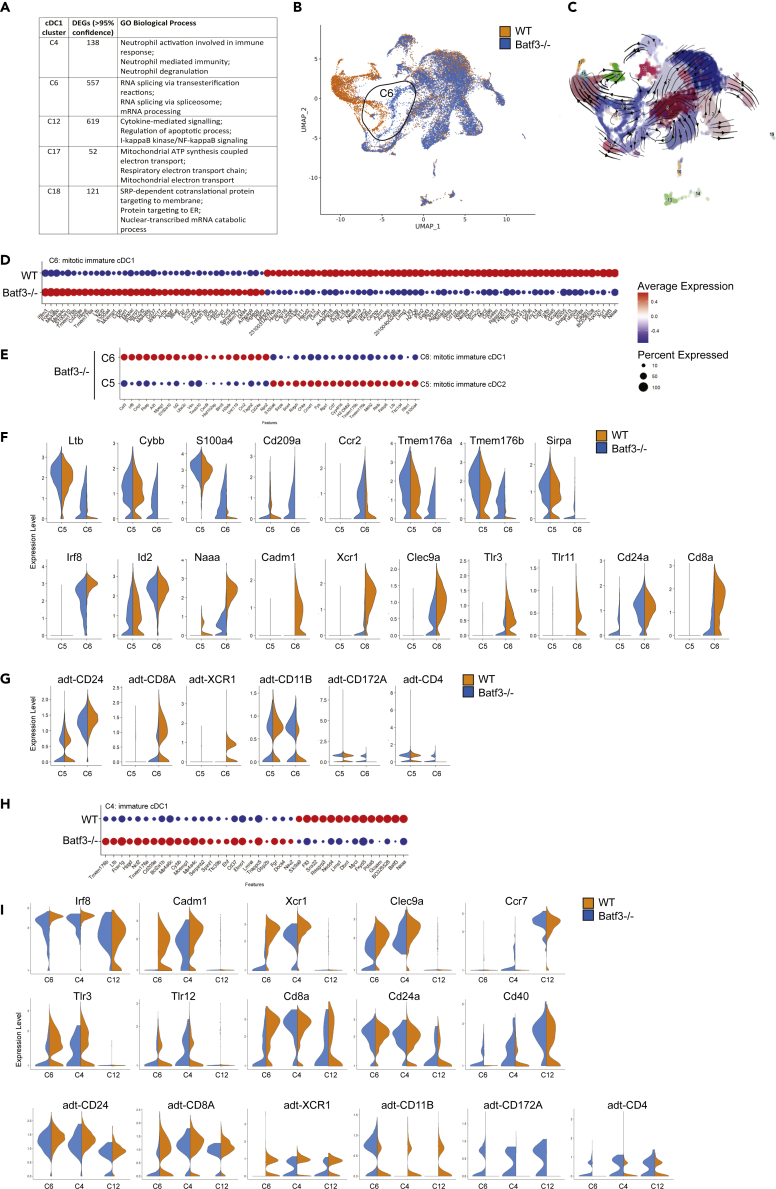
Residual mitotic cDC1-like cells in *Batf3*−/− mice increase expression of cDC2 features (A) GO analysis of cDC1 clusters C4, C6, C12, C17, and C18 (see Table S7). (B) Distribution of cells assigned to WT and *Batf3*−/− genotypes. (C) Trajectory analysis using RNA velocity. (D) DEGs between WT and *Batf3*−/− of cDC1 cluster C6 (see Table S1). (E) Top 40 DEGs between *Batf3*−/− mitotic cDC1 state C6 and *Batf3*−/− mitotic cDC2 state C5. (F) Gene expression of canonical cDC2 and cDC1 features between mitotic cDC1 state C6 and mitotic cDC2 state C5 (related to Figure S5). (G) Protein expression (adt) of canonical cDC2 and cDC1 features between mitotic cDC1 state C6 and mitotic cDC2 state C5. (H) DEGs between WT and *Batf3*−/− of cDC1 cluster C4 (see Table S1). (I) Gene and protein expression of canonical cDC1 features between mitotic cDC1 (C6), immature cDC1 (C4), and mature cDC1 (C12).

Batf3 affects the development and maintainace of cDC1 cells, and we therefore explored the cDC1 landscape between WT and *Batf3*−/− mice. The cDC1 landscape was represented in three major clusters C4, C6, and C12 (Figures 2D and 2E) and two minor clusters C17 and C18 enriched in mitochondrial and ribosomal genes, respectively (Figure S3C). To gain more insight into the differences between cDC1 cell states, we analyzed DEGs between all five cDC1 clusters (Table S7). GO analysis of DEGs suggested that C4 was enriched in gene expression associated with neutrophil responses, whereas C6 was associated with RNA splicing, and C12 with cytokine signaling and NF-κB signaling (Figure 5A).

cDC1 cluster C6 was equally represented by 797 WT and 727 *Batf3−/−* cells, but the two genotypes presented as distinct C6 populations suggesting genotype-based differences (Figure 5B). Functional enrichment analysis suggested that *Batf3−/−* C6 cells were clustered together with WT C6 cells based on the expression of genes related to mitochondrial inner membranes and nuclear chromosome parts, likely connected to their active cell cycle state (Table 1, Figure 2K, Table S3). The mitotic state of C6 cells suggests that these cells might represent an early trajectory to cDC1 cells, but most *Batf3−/−* C6 cells would not reach the cDC1 states represented by C4 and C12. RNA velocity analysis, a technique used to predict future cell states from the abundance of spliced and unspliced transcripts in single cells (La Manno et al., 2018; Bergen et al., 2020), revealed a distinct path from WT cluster 6 to cluster 4 and finally to cluster 12 (Figure 5C).

Differential expression analysis of C6 between WT and *Batf3*−/− cells identified 101 DEGs (adj. p value < 0.05), of which 62 genes were significantly downregulated in *Batf3*−/− cells and 39 genes were significantly upregulated (Figure 5D, Table S1). Downregulated genes in *Batf3*−/− C6 included *Naaa, Xcr1, Cadm1, Cd8a, Tlr3,* and *Tlr11* as cDC1-defining genes, and *Cxcl16, Cd81, Cd86,* and *Cd36* as genes related to maturation (Figure 5D); however, we did not observe specific GO or pathway-related enrichment. Upregulated genes in *Batf3*−/− C6 included *Ltb*, *Cybb* (NOX2), and *S100a4* as cDC2 features, and *Ccr2*, *Cd209a* (DC-SIGN), and *Tmem176a/b*, indicative of a cDC2a phenotype (Figure 5D). Noting that *Batf3*−/− C6 cells demonstrated downregulation of cDC1 features and upregulation of cDC2 features when compared with WT C6 cells, we analyzed DEGs between *Batf3*−/− C6 and the corresponding mitotic cDC2 cell state C5. Compared with classical mitotic cDC2 C5 cells, *Batf3*−/− C6 cells displayed increased levels of cDC1-defining genes and decreased levels of cDC2-defining genes (Figure 5E). By comparing the expression of cDC1 and cDC2 characteristic transcriptomic features between mitotic cDC2 cluster C5 and mitotic cDC1 cluster C6, and between genotypes, we demonstrate an intermediate phenotype of *Batf3*−/− mitotic cDC1 C6 cells based on their expression of most cDC1 and cDC2 (Figure 5F), and comparable results were obtained at protein level (Figure 5G). Interestingly, *Batf3−/−* C6 cells downregulated many cDC1 features but maintained comparable expression levels of *Cd24a* and *Id2* (Figure 5F). To analyze the distribution of cDC1 and cDC2 characteristic genes across *Batf3*−/− C6 cells, we paired (1) *Batf3*−/− C6 cells with WT C6 cells, revealing weak yet significant cDC2 gene expression compared with classical WT mitotic cDC1 cells or (2) *Batf3*−/− C6 cells with mitotic cDC2 C5 cells, which revealed weak yet significant cDC1 gene expression compared with classical mitotic cDC2 cells. The highest density of cDC1 and cDC2 genes was observed within the same *Batf3*−/− C6 cells (Figure S5). Our data suggest that mitotic early cDC1 cells might divert to the cDC2 lineage in the absence of Batf3. RNA velocity analysis predicted that mitotic *Batf3−/−* cDC1 (C6) cells either continued toward immature cDC1 cell state (C4) or followed a trajectory toward immature cDC2b (C2) (Figure 5C). It remains to be determined whether *Batf3*−/− mitotic cDC1 cells continue to increase expression of cDC2 features, resulting in a full convergence to cDC2, or abort a cDC2 trajectory altogether.

cDC1 cluster C4 identified 38 DEGs (adj. p value < 0.05) between genotypes, of which 14 were significantly downregulated in *Batf3*−/− cells, whereas 24 were significantly increased (Figure 5H, Table S1). Downregulated gene transcripts in *Batf3*−/− C4 included the cDC1-characteristic gene *Naaa*, whereas upregulated genes also included cDC2-characteristic genes *Ltb*, *Cybb* (NOX2), *Cd209a* (DC-SIGN), and *Tmem176a/b* (Figure 5H), similarly to those observed for C6. Of note, C4, predicted to be an immature cDC1 state when considering expression of activation features (Figure 2J), was represented by 2,202 WT cells but only 31 *Batf3−/−* cells, suggesting that *Batf3* is required to reach this immature cDC1 cell state, and that the absence of *Batf3* increases expression of cDC2 features.

Furthermore, cDC1 cluster C12 was represented by 394 WT cells but only 31 *Batf3−/−* cells, indicating that *Batf3* was also required for this cDC1 cell state. We identified 12 DEGs (adj. p value < 0.05) between genotypes, of which only *Batf3* was significantly downregulated in *Batf3*−/− cells, whereas expression of 11 genes was significantly increased (Table S1). These genes were, however, not associated with particular pathways and cell types. The expression of cDC1-characteristic genes *Cadm1*, *Xcr1*, *Clec9a*, *Tlr3,* and *Tlr11* was virtually absent in C12 cells, and *Irf8* expression was reduced (Figure 5I). C12 cells, however, expressed the highest levels of activation features *Ccr7, Cd40, Cd83, Fas,* and *Cd274* (PDL1) (Figures 2J and 5I), indicating that C12 represents mature cDC1 cells. Protein features CD24, CD8A, and XCR1 were maintained on C12 cells and were comparable to immature C4 cells (Figure 5I). DEGs of C12 cells were further enriched in TNF signaling (Table S7). Maintenance of XCR1 protein with downregulation of *Xcr1* gene expression on mature cDC1 has been reported by others (Ardouin et al., 2016; Crozat et al., 2011). DGE analysis between WT and *Batf3*−/− C12 cells suggested a high degree of similarity (Table S1), suggesting that the Batf3-dependent cDC1 trajectory follows an “abort/divert or proceed” principle.

### Characterization of *Sox4*+ cDCs

**Figure 6:**
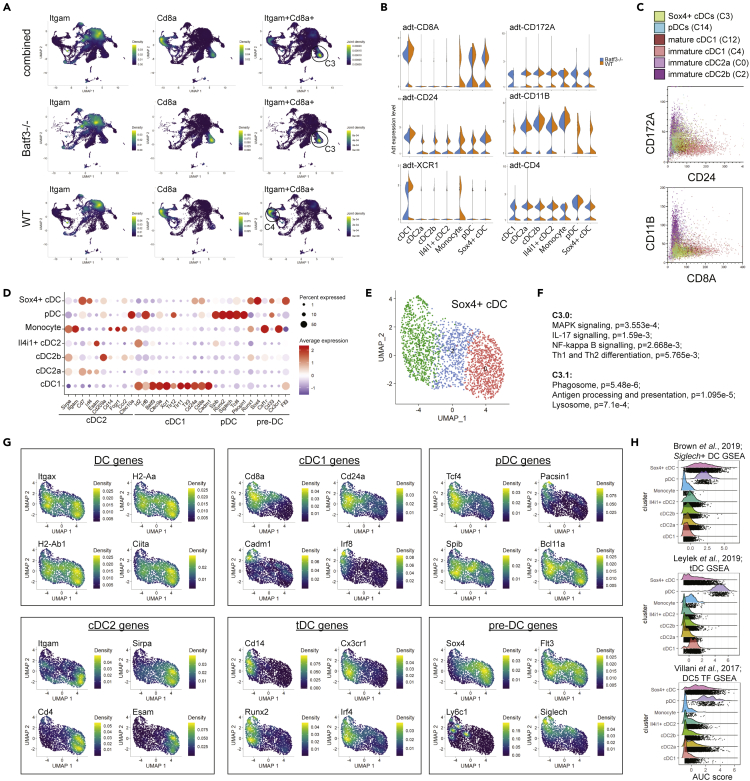
Characterization of a *Sox4*+ cDC cell state (A) Joint density of *Itgam* and *Cd8a*, using the Nebulosa package, identifying *Sox4*+ cDCs (C3) as cluster with co-expression in *Batf3*−/−. (B) Protein expression (adt signals) between metaclusters defined in Figure 3A. (C) Adt data of selected clusters were converted to FCS files and analyzed for co-expression of CD24 and CD172A or CD8A and CD11B using flow cytometric BD Kaluza software. (D) Expression of canonical DC genes between metaclusters. (E) Subclustering of *Sox4*+ cDCs (C3) using a resolution of 0.3. (F) GO pathway analysis of C3 subcluster-specific DEGs (see Table S8). (G) Distribution of canonical genes for DCs, pDCs, cDC1, cDC2, tDCs, and pre-DCs using the Nebulosa package. (H) GSEA using gene signatures from Brown et al. (2019); Leylek et al. (2019); Villani et al. (2017) and the AUCell package (see Table S2).

Given our observation that a small proportion of cDCs express both the cDC1 feature CD8 and the cDC2 feature CD11B, as described in Figure 1, we aimed to identify cells with these features in the sequencing dataset. We analyzed the joint density of *Cd8a* and *Itgam* using the *Nebulosa* package and found that the *Sox4*+ cDCs cluster (C3) was the only cluster expressing both features (Figure 6A). This joint signal was only observed in *Batf3*−/− *Sox4*+ cDCs, and not in WT *Sox4*+ cDCs (Figure 6A). Rather, WT DCs with a joint *Itgam/Cd8a* signal were observed in C4 immature cDC1 cells, although at reduced density (Figure 6A). The adt protein expression data showed that a proportion of *Sox4*+ cDCs expressed CD8A, CD24, CD172A, CD11B, and CD4 (Figure 6B). These data suggest that the CD8+CD11b+ cDCs described in Figure 1 might represent a mixture of cells, including *Sox4*+ cDCs and cDC1. We further assessed this using the adt-converted FCS files of each cluster using standard flow cytometry software, where we compared expression of CD11B versus CD8A and CD172A versus CD24 between selected cDC2 clusters (C0 and C2), cDC1 clusters (C4 and C12), pDCs (C14), and *Sox4+* cDCs (C3), confirming that a proportion of *Sox4*+ cDCs and cDC1 cells expressed CD24, CD8A, CD172A, and CD11B (Figure 6C).

DGE analysis of *Sox4*+ cDCs (C3) revealed *Batf3* as the only significant DEG between WT and *Batf3−/−* genotypes (Table S1), whereas DGE analysis between *Sox4*+ cDC (C3) and all other clusters and metaclusters revealed that C3 expressed key genes of several DC lineages including *Cd8a*, *Cd7, Siglech, Tcf4,* and *Spib* (Tables S1, S3, and S6, top 50 in Figure 3B). C3 expressed levels of MHCII genes comparable with other cDC clusters (Figure S3B). We compared the expression of key canonical DC genes between *Sox4*+ cDCs and cDC1, cDC2a, cDC2b, *Il4i1*+ cDC2, pDC, and monocytes (Figure 6D). *Sox4*+ cDCs expressed the highest level of *Irf4* among these clusters. Of note, compared withcDC1 cells, *Sox4*+ cDCs cells did not express cDC1-characteristic transcription factors such as *Irf8*, *Id2*, or functional genes *Xcr1*, *Clec9a, Tlr3,* or *Tlr11,* or only in a very small fraction of cells. *Sox4*+ cDCs cells expressed moderate levels of pDC canonical genes *Siglech*, *Spib*, *Tcf4, Bcl11a,* and *Runx2*, but also lacked expression of pDC-characteristic TLRs (Figures 2I and 2J). Instead, *Sox4*+ cDCs expressed high levels of *Sox4*, *Runx1*, and *Flt3*, which are also highly expressed by pre-DCs (Figure 6D). However, splenic pre-DCs are considered to be MHCII negative (Schlitzer et al., 2015), and we hypothesize that these cells might represent a cell state of pre-DC to cDC transition as described previously (Leylek et al., 2019). This cluster contained 2,245 cells, representing 9% of all DCs, and was enriched in *Batf3−/−* DCs (12.7%) compared with WT DCs (5.8%). Recent single-cell analyses described similar populations of *Siglech*+ DCs (Brown et al., 2019; Leylek et al., 2019) and suggested a possible link to a phenotype of circulating human AXL+ DCs (See et al., 2017, Villani et al., 2017; Alcantara-Hernandez et al., 2017).

The distribution of gene and protein expression within the *Sox4*+ cDCs cluster indicated additional cell state heterogeneity. We therefore subclustered C3 into three cell states (Figure 6E). DGE analysis revealed a significant number of DEGs between C3.0 and C3.1, but only two DEGs in C3.2, suggesting that C3.2 represents a mixture of C3.0 and C3.1 cells (Table S8). Pathway analysis determined that DEGs of C3.0 were enriched in pathways of MAPK signaling, IL-17 signaling, NF-κB signaling, and Th1/Th2 differentiation, whereas DEGs of C3.1 were enriched in pathways of phagocytosis and antigen presentation (Figure 6F), suggesting that C3.0 is more mature than C3.1. We determined the expression of DC lineage characteristic features across C3 cell states. The three C3 cell states expressed comparable levels of *Itgax* (CD11c) and MHCII genes (Figure 6G). Expression of pDC-characteristic features varied across cell states, with C3.1 expressing high levels of *Tcf4* and *Spib*, whereas *Bcl11a* was similarly expressed across all C3 cell states (Figure 6G). The highest levels of cDC1 features *Cd24a* and *Cd8a* were expressed in C3.1, showing a low to high expression gradient from C3.0 to C3.1 (Figure 6G). *Irf8* and *Cadm1* were expressed only in a very small proportion of C3.1 cells. In contrast, the highest levels of cDC2 features were expressed in C3.0, with a low to high expression gradient from C3.1 to C3.0 (Figure 6G). We further examined features of previously described “transitional” *Siglech*+ DCs (tDC) (Leylek et al., 2019), including *Cd14*, *Cxcr3, Irf4,* and *Runx2*, which partly overlap with cDC2b cells and had the highest expression in C3.1 (Figure 6G). Last, we examined features characteristic to pre-DCs. We observed that, whereas *Flt3* was similarly expressed across C3 cell states, *Sox4* had the highest expression in C3.0, but also lacked *Ly6c1* expression (Figure 6G). Gene set enrichment analysis (GSEA) determined that the *Sox4*+ cDC cluster was aligned with *Siglech*+ cDCs (Brown et al., 2019) and tDCs (Leylek et al., 2019), as well as with a transcription factor signature of AXL+ human cDCs (Villani et al., 2017) (Figure 6H, Table S2).

Our data revealed that splenic CD11c+ MHCII+ DCs contain at least two cell states with lineage mixed features, which share expression of *Bcl11a, Spib,* and *Flt3*, but co-express either *Tcf4, Cd8a, Cd24a, Cd14, Irf4, Cx3cr1, Runx2* and *Siglech*, or *Itgam, Sirpa, Cd4, Esam,* and *Sox4* (Figure 6G). However, these cell states are connected via cells expressing moderate levels of these features, indicating cell state plasticity.

**Figure 7:**
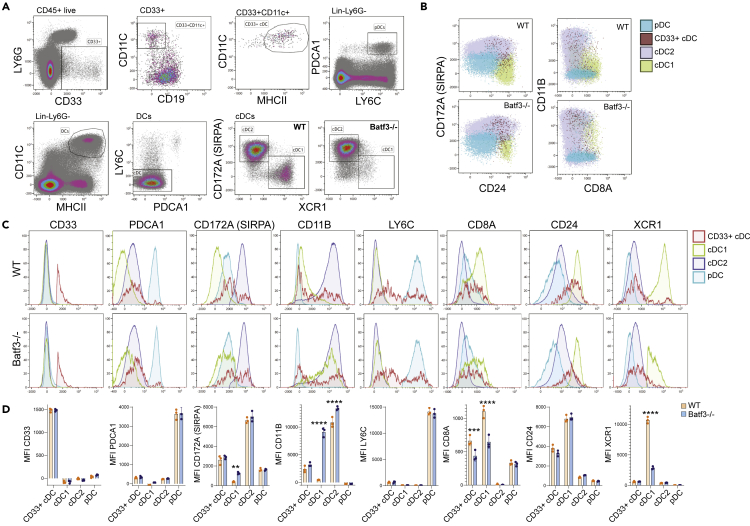
Immunophenotyping of CD33+ DCs Splenocytes of WT (n = 3) and *Batf3*−/− (n = 3) were incubated with a panel of 14 fluorochrome-conjugated antibodies. (A) CD33+ cDCs were gated as singlet live CD45+ LY6G− CD11C+ MHCII+. pDCs were gated as Lin− LY6G− PDCA1+ LY6C+. cDCs were gated as Lin− LY6G− CD11C+ MHCII+ LY6C−, and from these cDC1 and cDC2 were gated as XCR1+ or CD172A+, respectively. (B and C) Overlays of pDCs, cDC1, cDC2, and CD33+ cDCs. (D) Median fluorescence intensity (MFI) of cDC features between WT and *Batf3−/−* cDC1, cDC2, pDCs, and CD33+ cDCs. Each data point represents data of individual animal with mean ± standard error of the mean (SEM). Statistical significance was determined using unpaired t test with Welch correction. ∗∗p < v0.01, ∗∗∗p < 0.001, ∗∗∗∗p < 0.0001.

To verify whether *Sox4*+ cDCs contribute to the lineage-mixed phenotype observed in Figure 1, we analyzed splenic cDCs of WT and *Batf3*−/− mice with an extended flow cytometry panel. Expression of *Cd33* was largely unique to pDCs and *Sox4*+ cDCs (Figures 2I and 3B). We thus compared expression of common cDC features across cDC1, cDC2, CD33+ cDCs, and pDCs (Figures 7A–7D). CD33+ cDCs were distinct from pDCs but overlapped with both cDC1 and cDC2 in both WT and *Batf3*−/− cDCs with regard to expression of CD172A, CD11B, CD24, and CD8A (Figures 7A and 7B). CD33+ cDCs contributed to a population of cells expressing CD172A, CD11B, CD24, and CD8A, but did not represent the whole population (Figures 7A and 7B). In line with this, the transcriptomic data suggested that less than 50% of *Sox4*+ cDCs expressed *Cd33*, and hence other identifying surface features will be required to investigate this population in detail. Flow cytometry analysis confirmed that CD33+ cDCs largely lacked expression of the pDC feature PDCA1 (Figure 7C). All CD33+ cDCs expressed increased levels of CD24 and CD8A compared with cDC2 cells and pDCs, and decreased levels compared with cDC1, but largely lacked expression of XCR1 (Figure 7C). Expression of CD11B, CD172A, and LY6C on CD33+ cDCs was biphasic (Figure 7C), suggesting heterogeneity of two cell states as observed in *Sox4*+ cDCs of the transcriptomic data. The flow cytometric analysis further revealed that *Batf3*−/− cDC1 cells had a significantly increased expression of CD172A and CD11B, whereas expression of CD8A and XCR1 was decreased and CD24 was unchanged (Figure 7D). We further confirmed that a small proportion of WT cDC1 expressed CD11B (Figure 7B, C).

Combined, these data confirm that cDCs with lineage-intermediate phenotypes with regard to expression of CD11B, CD8A, and CD24 can represent either a fraction of cDC1 cells or a population of CD33+ cDCs, whose functionality remains to be determined.

## Discussion

We previously identified that in the absence of Batf3, a proportion of cDCs displays a lineage-intermediate phenotype defined by co-expression of CD11B, CD172A, and CD8A (Chandra et al., 2017). In the present study, we observed cDCs with a lineage-intermediate phenotype, further differentiated by altered expression of classical cDC surface features. We implemented multi-modal single-cell transcriptome and epitope sequencing technology to characterize this cDC lineage-intermediate cell state in detail. We identified a significant proportion of cDCs with lineage-intermediate gene and protein expression signatures displaying characteristic features of cDC1, cDC2, and pDCs in both WT and *Batf3−/−* spleens. These cells lacked a transcriptional signature of maturity, but instead expressed high levels of *Sox4*, *Flt3,* and *Cd33*, features associated with a pre-DC phenotype. This population was enriched in the *Batf3−/−* cDC compartment compared with WT. Furthermore, *Batf3−/−* mice lacked classical cDC1 cells but harbored a population of mitotic immature cDC1-like cells expressing reduced levels of cDC1 genes but increased levels of cDC2 genes. *Batf3*−/− cDC1-like cells lacked expression of genes linked to cDC functionality including *Xcr1*, *Clec9a,* and cDC1-specific TLRs. Algorithms predicting developmental trajectories suggested that these *Batf3*−/− mitotic cDC1-like cells give rise to either cDC1 and cDC2 lineages.

Flow cytometric analysis of murine splenic DCs can delineate two main populations: cDC1 and cDC2 based on expression of XCR1/CADM1 and CD11B/CD172A, respectively (Leylek et al., 2019). With the advancement of high-dimensional single-cell technologies, recent work has redefined the comprehension of DC heterogeneity. Unsupervised clustering approaches based on principal-component analysis have enabled the identification of cell state heterogeneities beyond pure phenotypic delineation of DC subtypes. However, because current algorithms do not distinguish between cell subsets and cell states when dividing data into clusters based on differential gene expression, the cluster annotation and interpretation remains a challenge, especially within a lineage where a continuum of different cell states rather than distinct subtypes is to be expected (Bassler et al., 2019), and this model is important to consider when interpreting clustered data. Our data support this paradigm as we observe that certain features are expressed only in a fraction of certain clusters. The presented data provide a basis for analysis of the continuum of DC states in more detail and reveal, for example, a certain cluster containing multiple cell states shared by cDC1, cDC2, and pDCs.

Recent high-dimensional analysis of DCs has uncovered additional heterogeneity particularly in the cDC2 compartment. At least two distinct cDC2 populations have been suggested in human and mice in recent years. In mice, these populations are distinguished by expression of *Cd7/Dtx1/Ccr6* (cDC2a) and *Cd209a/Clec10a/Clec12a* (cDC2b) (Brown et al., 2019), and in human corresponding cells have been termed DC2 and DC3 (Cytlak et al., 2020; Bourdely et al., 2020; Bosteels et al., 2020; Dutertre et al., 2019). Additional minor populations of cDC2 cells have been described, such as *Siglech*+ cDC2 (Brown et al., 2019) or transitional cDCs (Leylek et al., 2019). Our murine splenic DC dataset provides support of this cDC2 heterogeneity. We identified populations of immature and mature cDC2a and cDC2b populations, as well as a *Il4i1*+ cDC2 population enriched in gene expression suggestive of an activated and inflammatory phenotype and distinguished through expression of *Tcf7*, with downregulated classical cDC2a and cDC2b features. It remains to be determined if and how this *Il4i1*+ cDC2 population is related to the other cDC2 populations.

We further identified a population of *Siglech*+ cDCs, which we termed *Sox4*+ cDCs based on its most DEGs. These *Sox4*+ cDCs expressed lineage-mixed features of cDC1, cDC2, pDCs, and pre-DCs and could be further divided in two substates. DC cell states combining lineage features of cDC1, cDC2, and pDCs have been described by others. Wylie et al. recently identified a CD8+XCR1− DC population, which expressed lineage-mixed gene signatures of pDCs, cDC1, and cDC2 (Wylie et al., 2018). They showed that these CD8+XCR1− DCs preferentially expressed TLR5 and TLR7, and a unique set of endocytic receptors, and their ability to induce T helper cell proliferation was comparable with cDC2. The *Sox4*+ cDC cell state described here expresses low levels of *Tlr5* gene, and no other TLR genes, suggesting that these cells are limited to respond to pathogen-associated stimuli. Furthermore, Bar-On et al. described a steady-state CD8+ CX3CR1+ non-canonical DC population, which is related to pDCs (Bar-On et al., 2010). In fact, the *Sox4*+ cDC cell state we describe here highly expressed *Cx3cr1* in one cell state. Although *Cx3cr1* is expressed by many leukocytes including monocytes, macrophages, and DCs, it is significantly more highly expressed by pre-DCs (Choi et al., 2019), and the herein described *Sox4*+ cDC cell state expressed elevated levels of *Cx3cr1* and other pre-DC features such as *Flt3*, *Cd33,* and *Csf1r*, whereas any signs of maturity were absent. CCR2 was described as a required chemokine receptor to enable pre-DC migration (Nakano et al., 2017), and indeed the *Sox4*+ cDC cell state expressed increased levels of *Ccr2* compared with cDC1, cDC2a, and pDCs; however, CCR2 is also expressed by monocytes and the newly defined cDC2b subpopulation (Brown et al., 2019).

Schlitzer et al. described the transcriptional regulation of the MDP-to-CDP-to-pre-DC trajectory in detail and identified different pre-DC cell states that range from non-lineage primed to cDC1 or cDC2 lineage primed (Schlitzer et al., 2015). In spleen, Schlitzer et al. identified several pre-DC cell states distinguished by the expression of Ly6C, Ly6D, and SiglecH, and most of these cell states expressed moderate levels of CD24, but importantly these pre-DCs were CD11c+ MHCII−, whereas the *Sox4*+ cDC cell state described here expressed comparable levels of MHCII to other cDC1 and cDC2 cell states, but lacked expression of *Ly6c1*. We also found no evidence of enrichment of pre-DC gene signatures in *Sox4*+ cDCs using genes listed by Schlitzer (Schlitzer et al., 2015) (data not shown). Other recent studies also identified a DC cell state sharing cDC and pDC gene expression (Brown et al., 2019; Leylek et al., 2019; Alcantara-Hernandez et al., 2017), and GSEA determined that *Sox4*+ cDCs were similar to *Siglech*+ DCs and transitional DCs. An MHCII+ population expressing lineage-mixed features has also been identified in human blood and spleen (See et al., 2017). This population in humans expresses *Cd33*, *Cx3cr1*, *Cd327*, *Cd123*, *Cd5,* and *Cd2*. This population was shown to respond to CpG stimulation with secretion of TNFα and IL-12p40, and was able to induce CD4 T cell proliferation, whereas a pure pDC population was not. Furthermore, Villani et al. described a population of human DCs expressing *Axl* and *Siglec6* (annotated as “AS DCs”), which expressed a continuum of cDC and pDC lineage-mixed gene signatures across a trajectory prediction and were able to induce T cell proliferation (Villani et al., 2017). We showed that the murine *Sox4*+ cDC cluster and the human “AS DCs” had a similar transcription factor expression profile, which was also shared with pDCs. It has been suggested that “AS DCs” are in fact aligned with the pre-DC population recently described by others (Amon et al., 2020), and Brown et al. showed that adoptive transfer of *Siglech*+ DCs leads to development of both cDC1 and cDC2 cells (Brown et al., 2019).

The simplified approach to classify cDC1 as CD8+ or CD103+ in mice is insufficient to predict cross-presenting function. Although not all CD8+ or CD103+ DCs possess cross-presentation ability (Kanazawa, 2007), the expression of XCR1 appears to be an exclusive cross-presentation feature (Bachem et al., 2012; Guilliams et al., 2016). *Batf3*−/− mice are considered to lack cDC1 cells, but maintain a residual population of CD8+ cDCs. Our analysis determined that whereas *Batf3−/−* mice retained expression of CD8 and CD24 on cDC1-like cells, their expression of *Xcr1*/XCR1 and *Clec9a* was strongly diminished, although still elevated compared with classical cDC2 cells. These data suggest that the cDC1-like cells in *Batf3−/−* mice potentially retain suboptimal cross-presentation abilities, and this could explain some of the discrepancies described in the literature with respect to *Batf3*. We have previously reported that *Batf3−/−* mice were capable of rejecting neoantigen-expressing skin grafts, whereas a different mouse model lacking cDC1 cells (CD11cCre-Id2^flox/flox^) was unable to do so (Chandra et al., 2017). Our interpretation of these data was that the residual population of CD8+ cDCs in *Batf3−/−* mice retained functionality to enable skin graft rejection. Intriguingly, in the same study, we showed that *Batf3−/−* mice were unable to mount a delayed hypersensitivity response and phagocytose dying cells, highlighting a threshold effect determining functions that are retained in *Batf3−/−* cDC1-like cells and others that are lost. Of note, the cDC1 compartment can be completely restored in *Batf3−/−* mice through bacterial infection or administration of IL-12, in which case Batf compensates for the lack of Batf3 (Tussiwand et al., 2012). This implies that the ability for *Batf3*−/− mice to mount a cDC1-dependent immune response is affected by the housing conditions of the animals, where *Batf3−/−* mice display more severe immunodeficiency when housed in a very clean facility (Mott et al., 2015). Similar to *Batf3−/−* mice, Etv6 deficiency also leads to a diminished cDC1 compartment with limited functionality. However, whereas *Batf3−/−* cDC1-like cells upregulated cDC2 features, *Etv6−/−* cDC1-like cells increased gene signatures of pDCs (Lau et al., 2018). Together these data support a hypothesis of phenotypic DC plasticity, and future studies are required to identify drivers of such plasticity.

With the extension to multimodal technologies including the incorporation of epitope-specific antibodies, holistic analysis of cell states can overcome some of the limitations associated with undetectable transcript expression and dropouts in unimodal scRNA-seq data. However, integration of epitope and transcript analysis highlights the need for caution when using barcoded antibodies, and verification of specific antibody staining and setting of thresholds is crucial. The need for antibody validation might pose a limitation when using extensive antibody panels in a range of hundreds as attempted by others. From an antibody panel of 10, we excluded 3 where specific binding was not clear. The current study also observed scenarios where transcript expression was better detected than epitopes (e.g., *Sirpa*/CD172A and *Cd4*/CD4), and hence these antibodies did not add value to the analysis per se, but do act to support previous protein-based findings. This study provides the first CITE-seq dataset of murine splenic DCs of WT and *Batf3−/−* mice, which can serve to answer a variety of questions regarding steady-state DC heterogeneity and provides a road map to analyze DC cell state heterogeneity under inflammatory conditions in future studies.

### Limitations of the study

Precise classification of dendritic cell populations has been proved to be a challenging task for the field. Although DCs can be broadly classified by cell surface protein expression and the expression of specific transcription factors, this approach is often insufficient for cell populations where the main distinguishing features are shared or are dynamically expressed. This is clear in the case of cells undergoing lineage conversion and/or differentiation. Furthermore, current clustering approaches cannot readily distinguish similar cell subtypes and cell states. This is visible in our data, where the identity of certain *Sirpa*+ cDCs as cell type or cell state remains elusive (e.g., *Il4i1*+ cDC and *Sox4*+ cDCs). To determine functional differences between these cDC cell states or cell subtypes would require extensive *in vitro* efforts. However, surface features exclusively expressed by these cells were not identifiable, making their isolation for further experiments not possible.

In this study, we combined high-resolution single-cell gene expression profiling with cell surface protein expression to track DC-specific features in the absence of a crucial DC transcription factor, Batf3. Despite the high resolution and throughput afforded by droplet-based scRNA-seq, the sensitivity of gene detection is limited to capturing only a portion of the transcriptome, in terms of numbers of genes detected and gene biotype. This is largely due to the short region that is sequenced using current 3′ and 5′ methods, and, for now, this limits the detection of population-specific transcript isoforms. Furthermore, current methods are designed to capture poly-A-tailed transcripts; developments in whole transcriptome profiling that capture a wider range of transcription products may reveal new and interesting markers.

One of the main limitations of CITE-seq is the potential for high levels of antibody background signal, which may be attributed to non-specific binding or high staining concentration. Although highly expressed proteins provide clear signals that enable robust cell type discrimination, classifying populations with low levels of expression remains a challenge. Our study was also limited to detecting proteins expressed at the cell surface; future techniques that also measure internal protein expression levels will allow investigation of protein families such as transcription factors. In addition, future studies in this direction would benefit from extensive antibody titration to achieve the best signal-to-noise ratio, because concentrations determined previously by other methods including flow cytometry may not be instructive.

### Resource availability

#### Lead contact

Information and requests for resources may be directed to and will be fulfilled by the lead contact Janin Chandra (j.chandra@uq.edu.au).

#### Materials availability

This study did not generate new unique reagents.

#### Data and code availability

The Gene Expression Omnibus (GEO) accession number for the sequencing data reported in this article is GEO: GSE149544 (https://www.ncbi.nlm.nih.gov/geo/query/acc.cgi?acc=GSE149544).

## Methods

All methods can be found in the accompanying transparent methods supplemental file.

## Supplemental information

Document S1. Transparent methods and Figures S1–S5
