# Supplementary material for: Absence of Batf3 reveals a new dimension of cell state heterogeneity within conventional dendritic cells
Source: iScience. 2021 Apr 15;24(5):102402. doi: 10.1016/j.isci.2021.102402 (PMC8105636; doi:10.1016/j.isci.2021.102402)
Supplement: Document S1. Transparent methods and Figures S1–S5 [file mmc1.pdf]

iScience, Volume *24*

Supplemental information

Absence of Batf3 reveals a new dimension of cell state heterogeneity within conventional dendritic cells

Samuel W. Lukowski, Inga Rødahl, Samuel Kelly, Meihua Yu, James Gotley, Chenhao Zhou, Susan Millard, Stacey B. Andersen, Angelika N. Christ, Gabrielle Belz, Ian H. Frazer, and Janin Chandra

SUPPLEMENTAL FIGURES

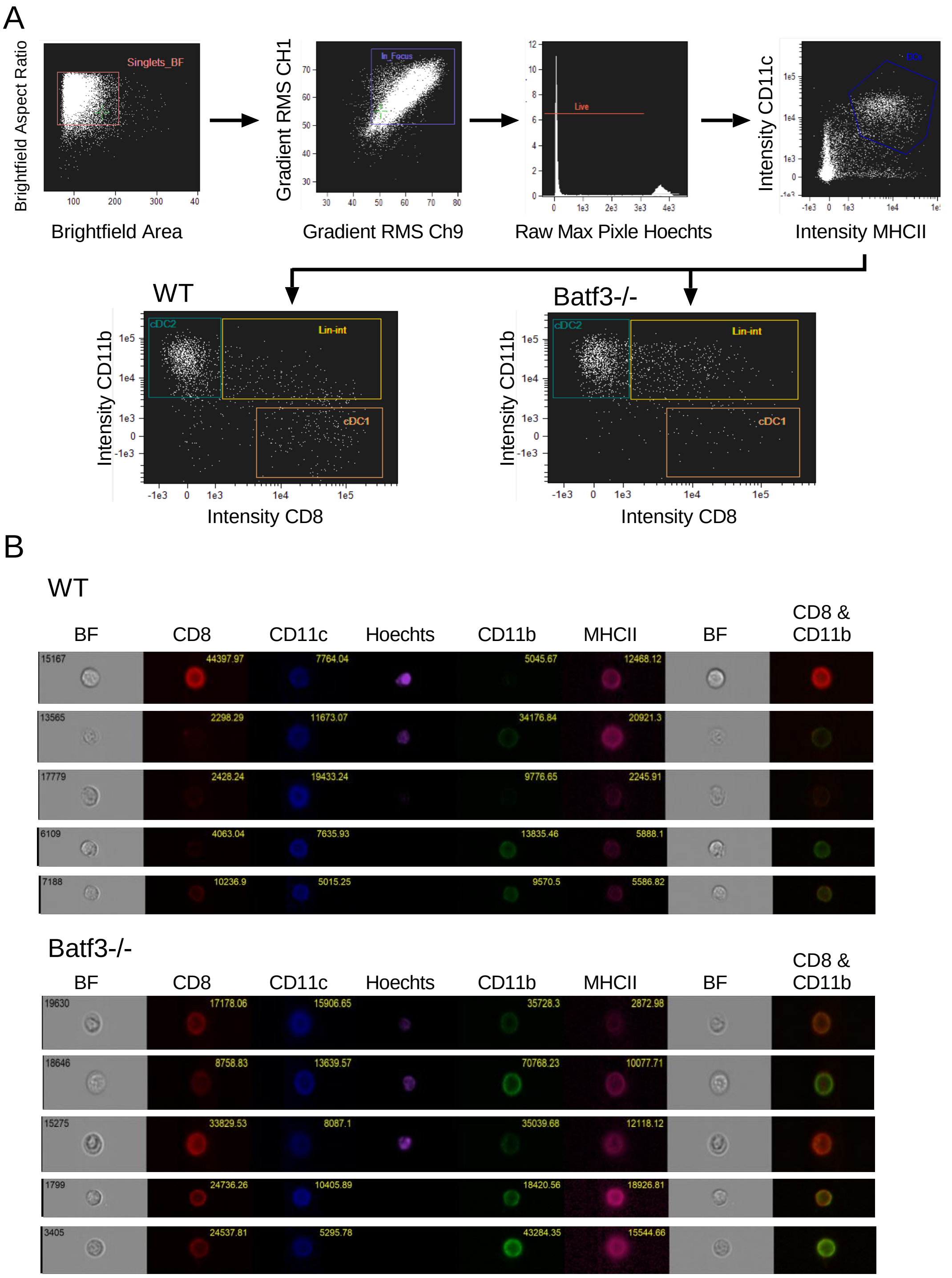

Figure S1. Identification of cDCs co-expressing CD8 and CD11b, related to Figure 1. Splenocytes of C57BL/6 (WT) and *Batf3-/-* mice were T and B cell depleted and labelled with Hoechst stain and antibodies specific to MHCII, CD11c, CD8 and CD11b before acquisition by imaging flow cytometry. **(A)** CD11c+ MHCII+ DCs were pre-gated from Hoechst negative, in-focus singlets. The DCs subsets were further defined as CD11b- CD8+ cDC1s, CD11b+ CD8- cDC2s, and CD11b+ CD8+ lineage-intermediate (lin-int) cDCs. **(B)** Examples of CD11b, CD8, MHCII, and CD11c expression of lin-int cDCs. BF; brightfield.

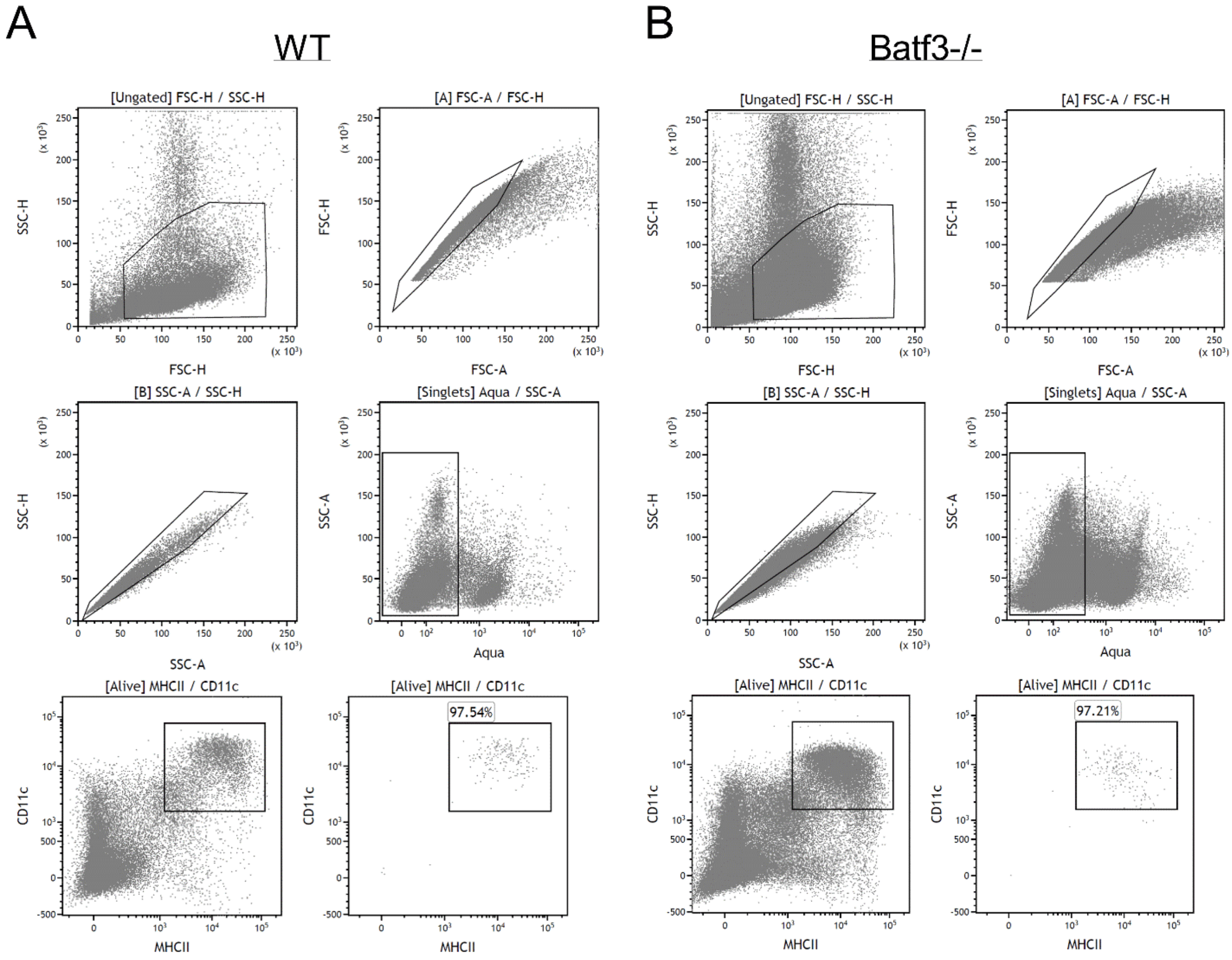

Figure S2. Sorting purities of cDCs, related to Figure 2. Alive (live-dead stain aqua negative) single MHCII+CD11c+ cells were sorted from 6 C57BL/6 (WT) and 6 *Batf3-/-* T/B cell depleted splenocytes. Purity of the sorted cells was analysed and depicted >97%.

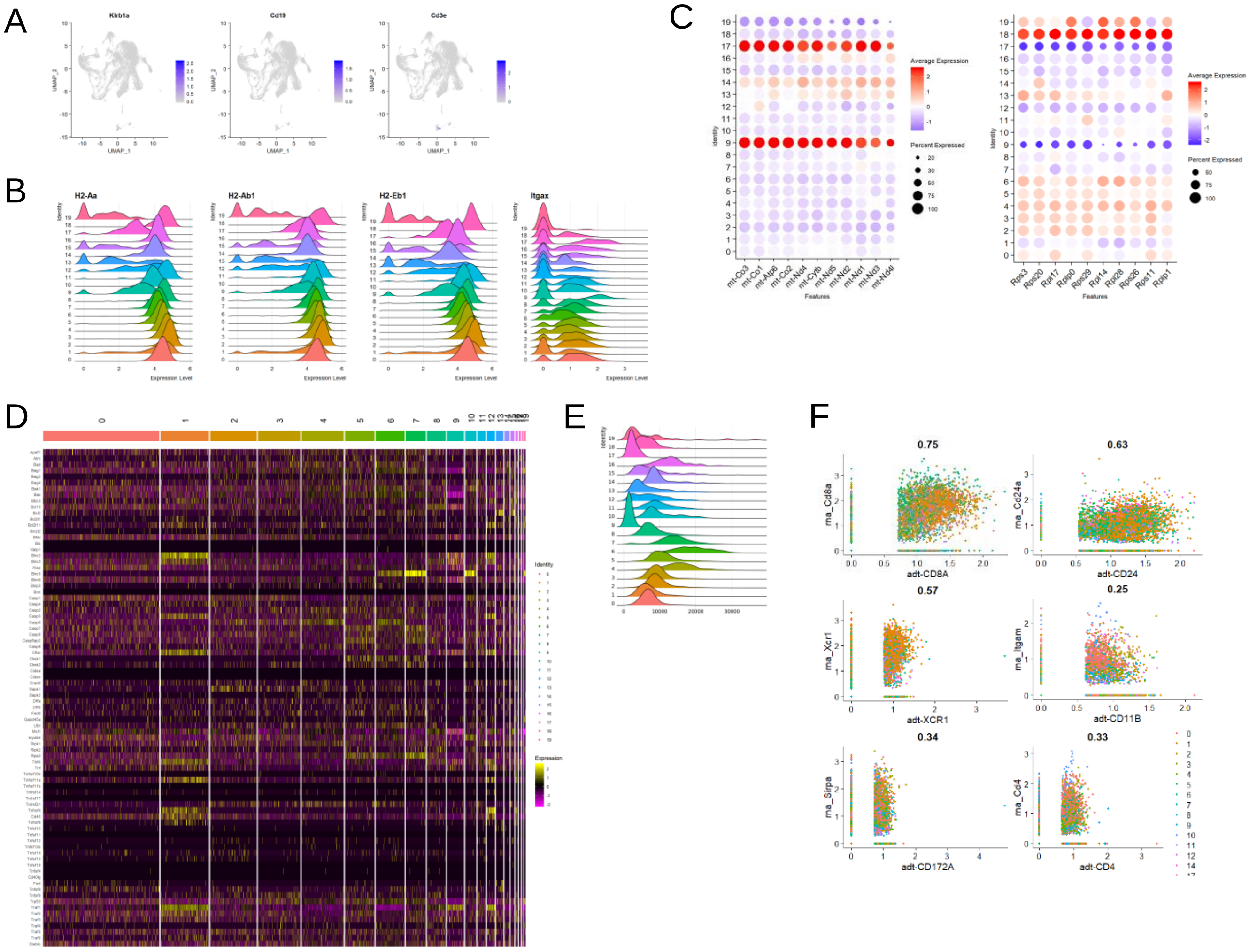

Figure S3. Additional single cell RNA sequencing information, related to Figure 2 and Figure 4. (A) Featureplots of *Klrb1a*, *Cd19* and *Cd3e* to determine contamination by NK, B or T cells. **(B)** Ridgeplots of MHCII genes and *Itgax* (CD11c) across clusters. **(C)** Expression of mitochondrial and ribosomal subunit gene transcripts. **(D)** Heatmap of expression of apoptotic transcripts. **(E)** UMI counts across clusters. **(F)** Pearson correlations between RNA and antibody-derived tags (adt).

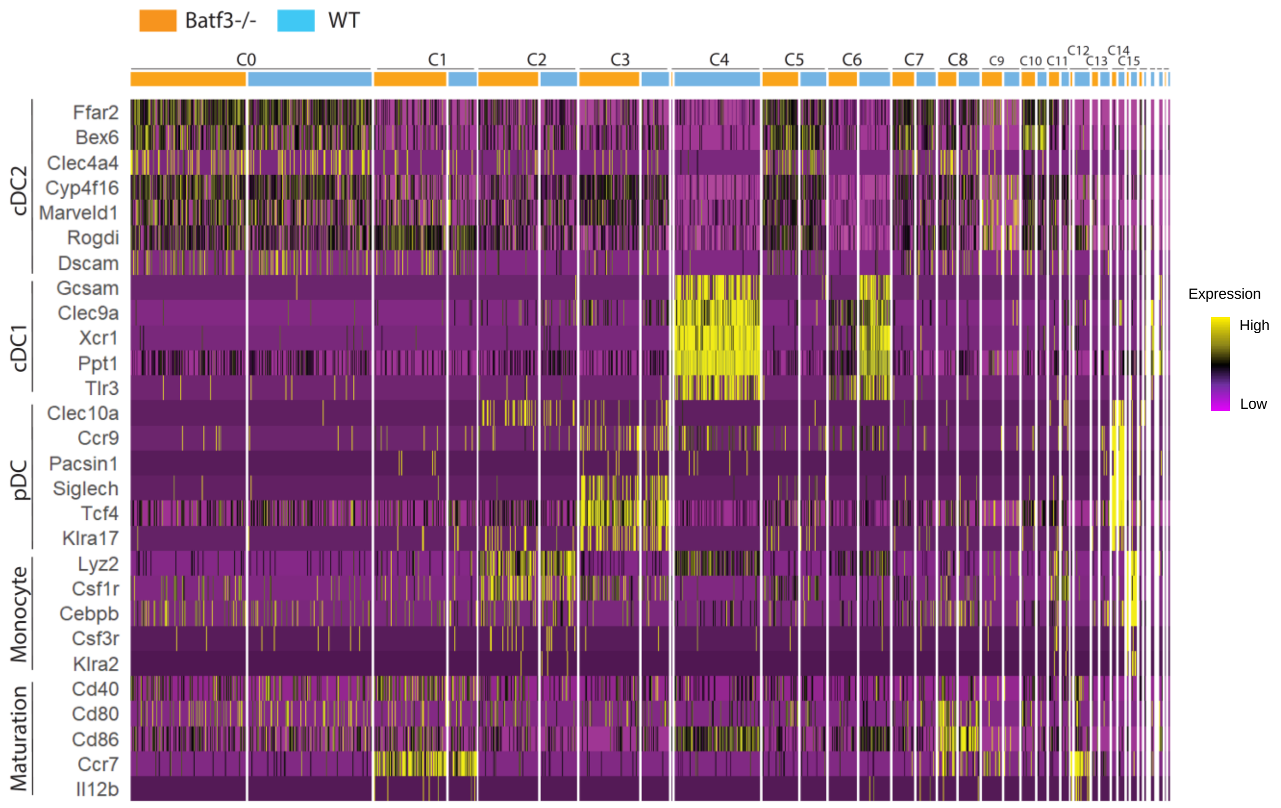

Figure S4. Expression of canonical DC genes across clusters, related to Figure 2. Heatmaps of canonical DC genes for WT (blue) and *Batf3-/-* (orange) clusters.

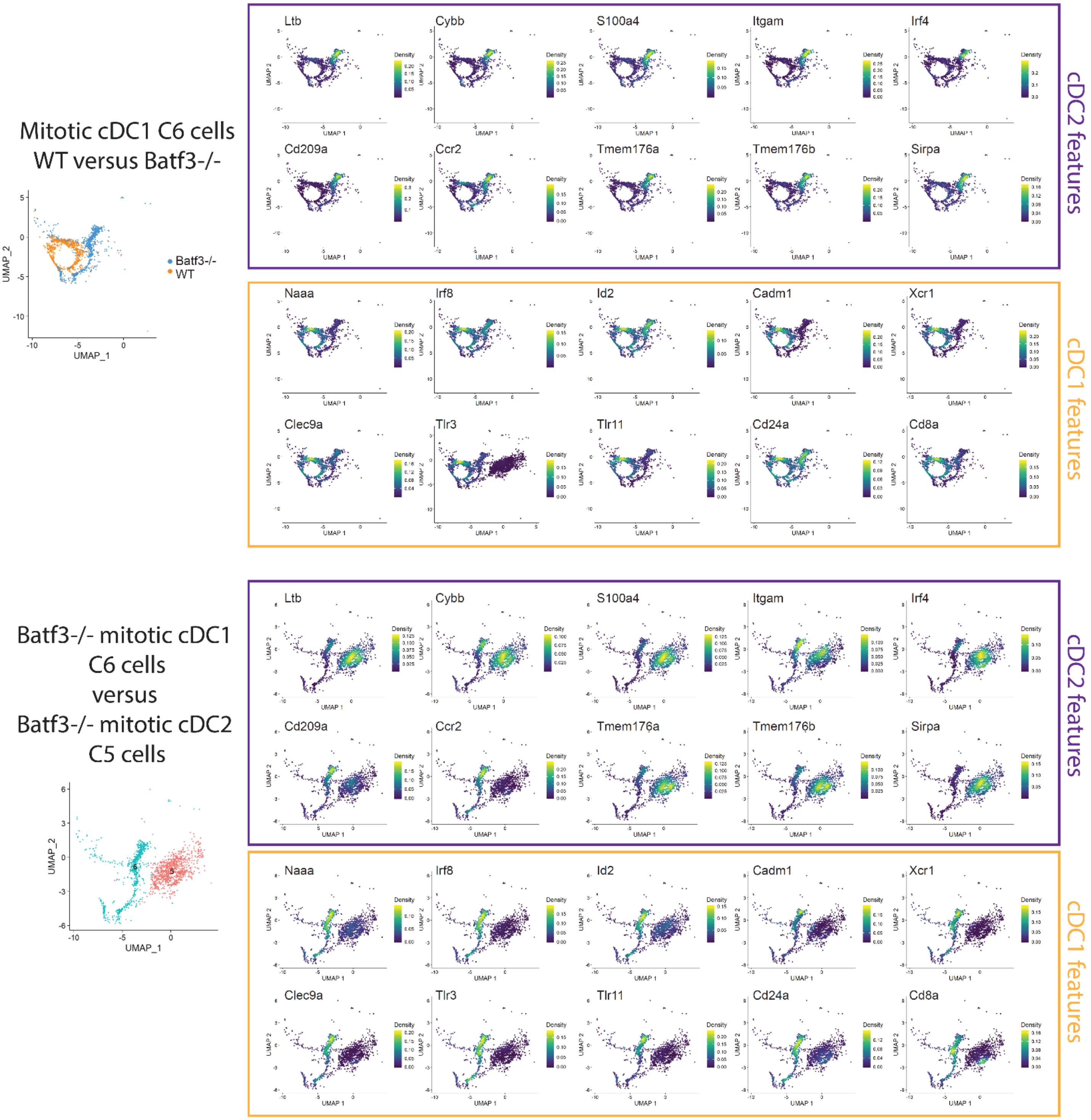

Figure S5. Residual mitotic cDC1-like cells in *Batf3*-/- mice increase expression of cDC2 features, related to Figure 5. Gene expression of cDC1 and cDC2 features was compared between WT and *Batf3*-/- of mitotic cDC1 (C6) cells (upper panels), or between *Batf3*-/- of mitotic cDC1 (C6) and *Batf3*-/- of mitotic cDC2 (C5) cells (lower panels).

TRANSPARENT METHODS

Key resources table

REAGENT or RESOURCE	SOURCE	IDENTIFIER
**Antibodies**		
Anti-mouse CD11c (PE-Dazzle)	Biolegend	Cat#117348; RRID: AB_2563655; Clone: N418
Anti-mouse I-A/I-E (APC-Cy7)	Biolegend	Cat#107628; RRID: AB_2069377; Clone: M5/114.15.2
Anti-mouse/human CD11b (Alexa Flour 700)	Biolegend	Cat#101222; RRID: AB_493705; Clone: M1/70
Anti-mouse CD8a (PE)	eBioscience	Cat#12-0081-82; RRID: AB_465530: Clone; 53-6.7
Anti-mouse/human CD11b (Brilliant violet 510)	Biolegend	Cat#101245; RRID: AB_2561390; Clone: M1/70
Anti-mouse TCRb (Biotin)	Biolegend	Cat#109204; RRID: AB_313427; Clone: H57-597
Anti-mouse CD19 (Biotin)	Biolegend	Cat#115504; RRID:

		AB_313639; Clone: 6D5
Anti-mouse CD45 (BUV615)	BD Biosciences	Cat#751170; RRID: AB_2875194; Clone: 30-F11
Anti-mouse CD3 (BV750)	Biolegend	Cat# 100225; RRID: AB_10900444; Clone: 17A2
Anti-mouse CD19 (BV570)	Biolegend	Cat# 115535; RRID: AB_10933260; Clone: B4
Anti-mouse Ly6G (BUV737)	BD Biosciences	Cat# 741813; RRID: AB_2871151; Clone: 1A8
Anti-mouse PDCA1 (APC)	Biolegend	Cat# 127015; RRID: AB_1967101; Clone:927
Anti-mouse CD11b (APC-R700)	BD Biosciences	Cat# 564985; RRID: AB_2739033; Clone: M1/70
Anti-mouse Ly6C (FITC)	Biolegend	Cat# 128005; RRID: AB_1186134; Clone: HK1.4
Anti-mouse I-A/I-E (BV711)	Biolegend	Cat# 107643; RRID: AB_2565976;

		Clone: M5/114.15.2
Anti-mouse CD11c (BV421)	Biolegend	Cat# 117329; RRID: AB_10897814; Clone: N418
Anti-mouse CD24 (BV605)	Biolegend	Cat# 101827; RRID: AB_2563464; Clone: M1/69
Anti-mouse XCR1 (BV650)	Biolegend	Cat# 148220; RRID: AB_2566410; Clone: ZET
Anti-mouse CD33 (PE)	Thermo Fisher Scientific	Cat# 12-0331-80; RRID: AB_2637178; Clone: 9A11
Anti-mouse CD172A (PerCP eFluor710)	Thermo Fisher Scientific	Cat# 46-1721-82; RRID: AB_10804639; Clone: P84
Anti-mouse CD4 (BUV496)	BD Biosciences	Cat# 612952; RRID: AB_2813886; Clone: GK1.5
Anti-mouse CD11c (Totalseq-A0106, GTTATGGACGCTTGC)	Biolegend	Cat#: 117355; RRID: AB_2750352; Clone: N418
Anti-mouse/ human CD11b (Totalseq-A0014, TGAAGGCTCATTTGT)	Biolegend	Cat#: 101265; RRID:

		AB_2734152; Clone: M1/70
Anti-mouse CD172a (Totalseq-A0422, GATTCCCTTGTAGCA)	Biolegend	Cat#: 144033; RRID: AB_2800670; Clone: P84
Anti-mouse CD370 (Totalseq-A0556, AACTCAGTTGTGCCG)	Biolegend	Custom-built; Part #: 96461; Clone: 7H11
Anti-mouse CD4 (Totalseq-A001, AACAAGACCCTTGAG)	Biolegend	Cat#: 100569; RRID: AB_2749956; Clone: RM4-5
Anti-mouse CD8a (Totalseq-A002, TACCCGTAATAGCGT)	Biolegend	Cat#: 100773; RRID: AB_2734151; Clone: 53-6.7
Anti-mouse F4/80 (Totalseq-A0114, TTAACTTCAGCCCGT)	Biolegend	Cat#: 123153; RRID: AB_2749986; Clone: BM8
Anti-mouse Siglec H (Totalseq-A0119, CCGCACCTACATTAG)	Biolegend	Cat#: 129615; RRID: AB_2750537; Clone: 551
Anti-mouse, rat XCR1 (Totalseq-A0568, TCCATTACCCACGTT)	Biolegend	Cat#: 148227; RRID: AB_2783120; Clone: ZET
Anti-mouse CD24 (Totalseq-A0212, TATATCTTTGCCGCA)	Biolegend	Cat#: 101841; RRID: AB_2750380; Clone: M1/69

Anti-mouse CD117 (Totalseq-A0012, TGCATGTCATCGGTG)	Biolegend	Cat#: 105843; RRID: AB_2749960; Clone: 2B8
**Biological Samples**		
N/A		
**Chemicals, Peptides, and Recombinant Proteins**		
Collagenase D	Roche	11088858001
DNase	Roche	11284932001
**Critical Commercial Assays**		
EasySep™ Mouse Streptavidin RapidSpheres™ Isolation Kit	EasySep	Cat#19860
LIVE/DEAD™ Fixable Aqua Dead Cell Stain Kit	Invitrogen	Cat#L34965
Viability stain FVS	BD Biosciences	Cat#566332
**Deposited Data**		
GSE149544	NCBI GEO	
**Experimental Models: Cell Lines**		
N/A		
**Experimental Models: Organisms/Strains**		
C57BL/6	Animal Resources Centre	Product code: A2KB

Batf3-/-	Jackson Laboratories	Stock#013755
**Oligonucleotides**		
AF647-dT [5'-5Alex647N-T_{30}-3’]	IDT	
**Recombinant DNA**		
N/A		
**Software and Algorithms**		
IDEAS	Life Science Research	
Kaluza	Beckman Coulter	https://www.beckman.com.au/flow-cytometry/software/kaluza; RRID:SCR_016182;
GraphPad Prism	Prism version 7	https://www.graphpad.com/scientific-software/prism; RRID: SCR_002798
bcl2fastq	2.2.0	
Cellranger	3.0.2	
Seurat	3.0.5	
SingleR	1.0	
Nebulosa	0.99.92	

AUCell	1.12.0	
Velocyto	0.17.15	
scvelo	0.2.1	
**Other**		
N/A		

Experimental models and subject details

Mice

C57BL/6 mice (WT) were obtained from the Animal Resources Centre (Perth, Western Australia, Australia). *Batf3-/-* mice were purchased from Jackson Laboratories (Bar Harbor, ME, USA) and bred in house. All mice were kept under specific pathogen-free conditions at the Biological Research Facilities of Translational Research Institute. All animal procedures and experiments were performed in compliance with the ethical guidelines of the National Health and Medical Research Council of Australia, with approval from the IMVS Animal Ethics Committee and the University of Queensland Animal Ethics Committee (UQDI/452/1)

Method details

Cell isolations

Spleens were removed from CO_2-euthanised mice and injected with 0.5mg/ml collagenase D (Roche, 11088858001) and 0.1 mg/ml DNase (Roche, 11284932001) diluted in PBS 10% FCS for 30 min at 37° C. The tissue was mechanically disrupted by straining through a 70 mm cell strainer (BD Falcon) into 50 ml falcon tubes and washed with PBS to a total volume of 15 ml. Single cell suspensions were centrifuged at 350g for 7 min, followed by red blood cell lysis using ammonium chloride potassium (ACK) buffer for 2 min on ice and subsequent wash with PBS. Cell pellets were resuspended in 2.5 ml PBS buffer. Cells were counted using TC20 automated cell counter (Bio-Rad) by staining with trypan blue (Sigma-Aldrich) for dead cell discrimination.

T and B cell depletion

To enrich splenic dendritic cells, T and B cells were depleted from splenic single cell suspension using EasySep Mouse Streptavidin RapidSpheres (StemCell Technologies) in accordance to the manufacturer's protocol. Biotin conjugated antibodies used for depletion were TCRb (Biolegend; clone H57-597; dilution 1:200) and CD19 (Biolegend; clone 6D5; dilution 1:200). In detail 1 x 10^8 cells were resuspended in 1 ml FACS buffer in round bottomed 5 mL tubes (BD Falcon) and incubated with 5 µl of each antibody for 10 minutes at room temperature. 75 µl of RapidSpheres magnetic beads were added followed by 2.5 minutes of incubation. The tube was placed in an EasySep magnet (STEMCELL Technologies) and incubated for 2.5 minutes before the enriched cells were carefully decanted into a new tube.

Flow cytometry analysis

Approximately 1 million cells from splenic single cell suspension was added to 5 mL round bottomed tubes (BD Falcon) for unstained and single stain controls. For samples with multiple stains approximately 4 million cells were added to each tube. 1 ml of PBS was added to all tubes before centrifuging at 350g for 5 min. To exclude dead cells, samples were treated with live/dead stain (Invitrogen, dilution 1:250 or BD Bioscience 1:500) diluted in PBS for 20 minutes on ice followed by PBS wash and centrifugation. Cells were incubated with FC block (BD Pharmingen; dilution 1:100) at 4°C for 10 minutes. Cells were incubated with anti-mouse monoclonal antibodies in FACS buffer (2% HI FBS, 1x PBS, 2 mM EDTA) for 20 minutes on ice. Cells were washed with FACS buffer and centrifuged, followed by resuspension in 400 µl FACS buffer. BD LSR Fortessa X20 and BD Symphony flow cytometers were used to acquire cells and Kaluza software (Beckman Coulter) was used for compensation and analysis.

Imaging flow cytometry

After T and B cell depletion the cells were incubated with Hoechst dye and FC block (BD Pharmingen; dilution 1:100) at 4°C for 10 minutes. mAbs against CD11c (Clone: N418; dilution 1:50), MHCII (Clone: M5/114.15.2; dilution 1:100), CD11b (Clone: M1/70; dilution 1:50), and CD8 (Clone; 53-6.7; dilution: 1:100) were used and the samples were incubated at 4°C for 30 minutes. The cells were acquired on an AMNIS ImageStreamX MkII (Luminex Corporation) and analysed using IDEAS software (Life Science Research).

Flow cytometric activated cell sorting

For CITE sequencing, 2 x 10^8 of pooled splenocytes from six C57BL/6 and six *Batf3-/-* mice respectively were resuspended in 1 mL FACS buffer and used for downstream analysis. Following T and B cell depletion (described above) cells were incubated with FC block (BD Pharmingen; dilution 1:100) simultaneously with live-dead Aqua stain (Invitrogen, dilution 1:250). Cells were subsequently stained with anti-mouse monoclonal antibodies against MHCII and CD11c in accordance with the staining protocol described above. Live $MHCII^+CD11c^+$ cells from each genotype were sorted into 1.5ml tubes (Eppendorf) containing 500µl 100% FCS using droplet-based sorters. Sorted cells were centrifuged at 350g for 5 minutes.

Incubation of barcoded antibodies

Sorted DC cell pellets were resuspended in 50 µl FACS buffer and incubated with 5 µl FC block for 10 minutes on ice, followed by PBS wash and centrifugation. We added 0.5 µg of each TotalSeq-A antibody (Biolegend) as an antibody pool to each of the samples. Cells were transferred to 1.5 mL tubes (Eppendorf) and incubated for 30 minutes on ice, followed by wash using 2% BSA/ 0.01%

Tween in PBS and centrifugation at 350g for 5 minutes at 4°C. DCs were resuspended in FACS buffer, cell viability and numbers were estimated by manual counting using trypan blue.

Single cell RNA sequencing

Sample preparation and 10x Chromium pipeline was performed in accordance with the manufacturer's protocol. To target 12,500 cells per sample ~25 000 cells of each genotype were loaded onto the chip for Chromium Single Cell Gel Bead and Library Kit v3 (10x Genomics) to account for a ~50% cell loss during microfluidics processing. The 10x Genomics User Guide was followed for GEM generation and barcoding, cDNA amplification and library construction. Eleven cDNA amplification cycles were performed, and one quarter of the cDNA was used as input for library construction. Library construction for CITE-seq was conducted in accordance with Totalseq-A antibody manufacturer protocol (Biolegend, v.3). Final indexing PCR was performed in a C1000 Touch thermal cycler with a Deep Well Reaction Module (Bio-Rad). For gene expression and CITE-seq libraries 11 and 10 SI-PCR cycles were used, respectively. Using the High Sensitivity DNA kit on Agilent BioAnalyser 2100 the libraries were quantified (Agilent, 5067-4626). Equimolar ratios were used to pool gene expression libraries, and of the total sequencing pool concentration 10% were spiked in cell hashing libraries. Quantification of the final pool was conducted by qPCR using KAPA Library Quantification Kit in combination with the Life Technologies Viia 7 real time PCR instrument (KAPA Biosystems, KK4824). Illumina NextSeq-500 was used for sequencing the denatured libraries using a 150 cycle High-Output Kit. This included Read 1 (28 bp) supplying the cell's barcode (16nt) and UMI (12nt), i7 (8bp) for sample index and Read 2 (111 bp) to capture the 3' sequence of the transcripts. In total, two sequencing runs were performed to obtain an average read depth of ~30,000 reads per cell.

<u>Quantification and statistical analysis</u>

Single cell RNA sequencing data analysis

The Illumina sequencing output was processed using the 10x Genomics Cell Ranger 3´ gene expression and feature barcoding pipeline (v3.0.2). Illumina bcl2fastq (v2.2.0) was used to demultiplex the raw Illumina reads and generate FASTQ files for individual RNA and ADT libraries. Cellranger count was used to map the sequencing reads to the GRCm38 (mm10) mouse reference genome, and the aggregate function was used to combine the libraries into a single matrix and correct for differences in sequencing depth. The aggregated gene expression and antibody count data, comprising wildtype and knockout libraries were further processed and analysed using Seurat (v3.0.5) (Stuart et al., 2019). Filtering and quality control were performed to remove outlier cells and

transcripts. Transcripts were retained if they were expressed in at least 3 cells, and a preliminary filtering of cells was done to remove cells with fewer than 200 transcripts. Further filtering was done based on number of transcripts in each cell with a lower threshold of 500 transcripts and the maximum number of transcripts per cell set to 5000. Cells with mitochondrial or ribosomal reads accounting for more than 20% and 50% respectively, were excluded from downstream analysis. Cell-cell gene expression variation was normalised using log transformation and the antibody counts were normalised using centered log-ratio (CLR), each followed by scaling as implemented in the Seurat pipeline. To reduce the dimensionality, we performed principal component analysis (PCA) and the cut-off was set to where an 'elbow' was observed in the curve. The first 16 PCs were used to construct a k-nearest neighbour graph using the FindNeighbors function in Seurat. Similar cells were then grouped together using the Louvain algorithm in the FindClusters function using the default resolution of 0.5 (Stuart et al., 2019). Cells were visualised in a two dimensional space using non-linear dimensional reduction via uniform manifold approximation and projection (UMAP) implemented in the Seurat pipeline.

Multi-modal cell type classification

Cell identities were first predicted by SingleR analysis using the Immunological Genome Project (ImmGen) database as a reference (https://github.com/dviraran/SingleR). Single cells and clusters were annotated based on gene expression markers of immune cell subsets described in ImmGen. Gene set enrichment analysis (GSEA) was performed using DC lineage-specific gene signatures outlined and referenced in Suppl. File 1 and using the AUCell package. Populations were further identified using known markers characteristic to cDC1, cDC2 and pre-cDCs. Unbiased population identification was performed by differential gene expression analysis. Using the FindAllMarkers algorithm implemented in Seurat, we obtained positive gene markers, expressed in at least 25% of the cells at a minimum of 0.25-fold difference (log-scale) between the cluster and the remainder of the dataset. The top 10 differentially expressed genes were visualised in a heatmap. Clusters were further distinguished by differential expression between specific populations and expression of DC lineage-specific gene markers. Cell cycle phase prediction was performed in Seurat using a dataset of cell-cycle-specific gene markers (Kowalczyk et al., 2015). Cells were assigned to the G2/M and S phases based on expression of gene markers associated to these phases. Cells not expressing markers of either G2/M or S phases were assigned to G1.

RNA velocity

Spliced and unspliced transcripts were identified for each cell using velocyto in combination with the position sorted BAM files produced by cellranger for the wildtype and *Batf3*$^{-/-}$ samples. The

individual velocity results for each sample were merged and the cell barcodes were matched to only contain the cell barcodes present in the Seurat object used for the main expression analysis. Next, a loom file was created from the full Seurat object, and the merged velocity data was processed and analysed using scvelo.

Protein expression profiles using oligo-tagged antibodies (CITE-seq)

To account for non-specific binding of the TotalSeq-A antibodies, we defined antibody-specific thresholds using flow cytometry analysis of the TotalSeq-A antibodies hybridised to a fluorochrome-conjugated oligo-dT probe and incubated with C57BL/6 splenocytes together with fluorochrome conjugated antibodies against CD11c and MHCII (Fig. S4B). After pre-selection of DCs, the percentage of positive staining of TotalSeq-A antibodies was translated into an adt bin value, which was used as cut-off point for the adt data. All values below the antibody-specific cut off point were set to zero. The bin-corresponding adt threshold value determined in C57BL/6 was subsequently applied as cut-off in the *Batf3-/-* data.

Statistical analysis

Analysis of data was performed using either unpaired two-tailed Student's t test or one-way ANOVA with Tukey's multi-comparison post-hoc test with a 95% confidence interval (Prism, GraphPad Software). *p<0.05; **p<0.01; ***p<0.001; ****p<0.0001. Information regarding number of replicates, sample size, significance test, value and meaning of n for each experiment are included in each figure legend. CITE-seq experiment was performed once.

<u>Data availability</u>

The accession number for the sequencing data reported in this paper is GSE149544.

SUPPLEMENTAL REFERENCES

KOWALCZYK, M. S., TIROSH, I., HECKL, D., RAO, T. N., DIXIT, A., HAAS, B. J., SCHNEIDER, R. K., WAGERS, A. J., EBERT, B. L. & REGEV, A. 2015. Single-cell RNA-seq reveals changes in cell cycle and differentiation programs upon aging of hematopoietic stem cells. *Genome Res,* 25**,** 1860-72.

STUART, T., BUTLER, A., HOFFMAN, P., HAFEMEISTER, C., PAPALEXI, E., MAUCK, W. M., 3RD, HAO, Y., STOECKIUS, M., SMIBERT, P. & SATIJA, R. 2019. Comprehensive Integration of Single-Cell Data. *Cell,* 177**,** 1888-1902 e21.
